# Effect of palmitoylation on the dimer formation of the human dopamine transporter

**DOI:** 10.1038/s41598-021-83374-y

**Published:** 2021-02-18

**Authors:** Talia Zeppelin, Kasper B. Pedersen, Nils A. Berglund, Xavier Periole, Birgit Schiøtt

**Affiliations:** 1 Department of Chemistry, Aarhus University, Aarhus C, Denmark; 2 Interdisciplinary Nanoscience Center, Aarhus University, Aarhus C, Denmark; 3 Present Address: School of Biological Sciences, University of Auckland & Canterbury, Auckland & Christchurch, New Zealand

**Keywords:** Biophysics, Computational biology and bioinformatics, Neuroscience, Chemistry

## Abstract

The human dopamine transporter (hDAT) is one in three members of the monoamine transporter family (MAT). hDAT is essential for regulating the dopamine concentration in the synaptic cleft through dopamine reuptake into the presynaptic neuron; thereby controlling hDAT dopamine signaling. Dysfunction of the transporter is linked to several psychiatric disorders. hDAT and the other MATs have been shown to form oligomers in the plasma membrane, but only limited data exists on which dimeric and higher order oligomeric states are accessible and energetically favorable. In this work, we present several probable dimer conformations using computational coarse-grained self-assembly simulations and assess the relative stability of the different dimer conformations using umbrella sampling replica exchange molecular dynamics. Overall, the dimer conformations primarily involve TM9 and/or TM11 and/or TM12 at the interface. Furthermore, we show that a palmitoyl group (palm) attached to hDAT on TM12 modifies the free energy of separation for interfaces involving TM12, suggesting that S-palmitoylation may change the relative abundance of dimers involving TM12 in a biological context. Finally, a comparison of the identified interfaces of hDAT and palmitoylated hDAT to the human serotonin transporter interfaces and the leucine transporter interface, suggests similar dimer conformations across these protein family.

## Introduction

**Figure 1:**
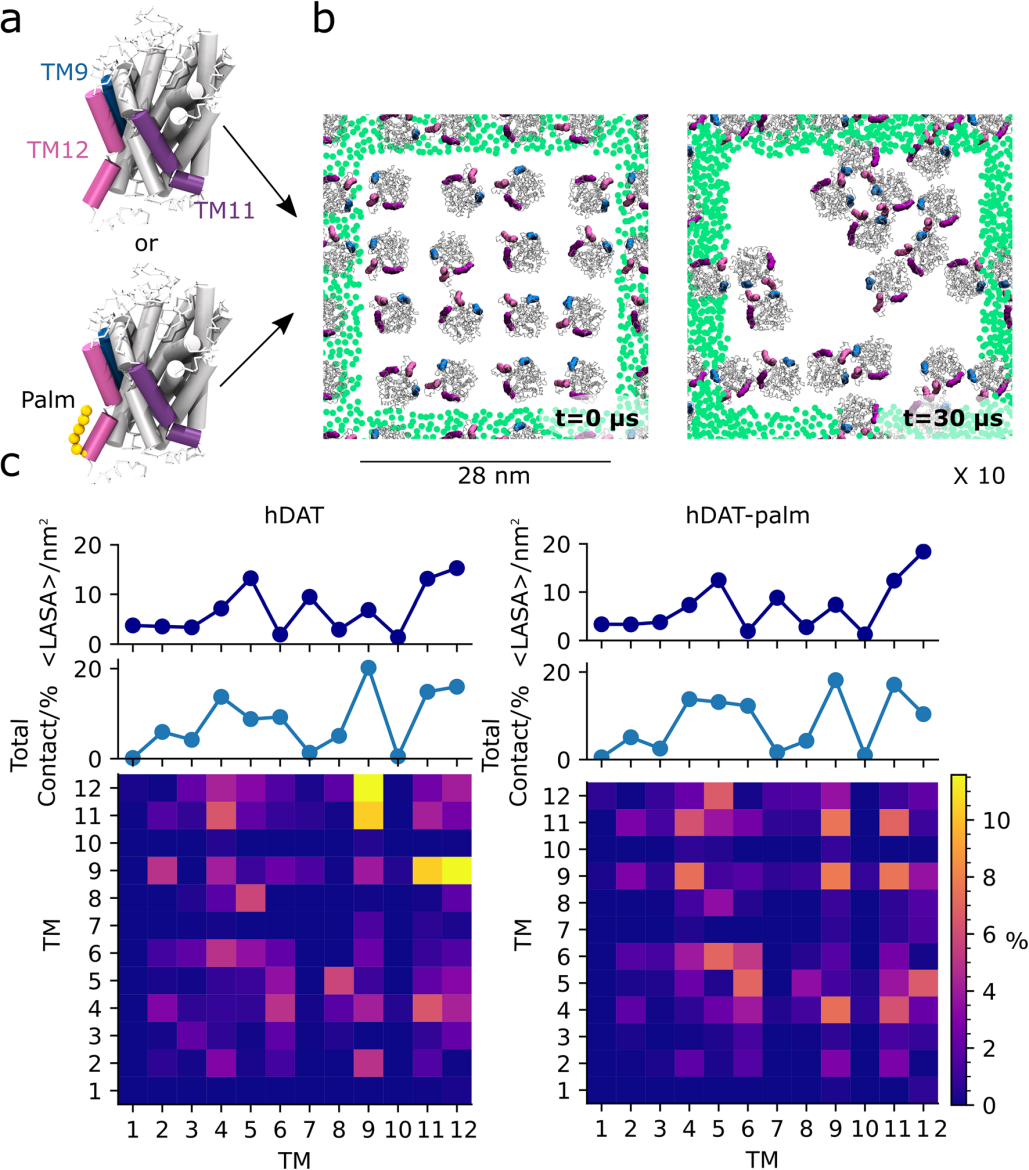
System setup and interhelical contact maps of hDAT and hDAT-palm excluding the palmitoyl group. (**a**) The hDAT CG structures with and without a palm group (orange spheres) attached to TM12. Shown in (**b**) is a top view of one of a system a(top view with respect to the membrane) at 0 µs (left) and after a 30 µs simulation (right). In the two systems 16 hDAT and hDAT-palm molecules were initially equally spaced and randomly orientated with respect to each other in a pure POPC membrane. hDAT and hDAT-palm were simulated for 30 µs and repeated 10 times. Palm, TM9, TM11, and TM12 are highlighted in orange, blue, purple, and magenta, respectively. The green dots represent the GL1 bead of POPC in the nearest periodic images for the system. Illustrated in (**c**), hDAT (left) and hDAT-palm (right), are the per helix contact maps calculated for all possible dimer pairs. An interhelical contact was considered when any residue in one protomer’s helix was within 7 Å of any residue in the other protomer’s helix (not considering the palm group). The contacts have been normalized by the total number of contacts calculated for the hDAT system. The 1D plot immediately above the contact map illustrates the sum of all TM contacts calculated for each helix given in %. The top 1D plot illustrates the average lipid accessible surface area in nm^2^ of the different helices in the two systems using a probe of size 0.26 nm. The standard deviations are not visible in the plots due to their small sizes (they vary between 0.23 and 0.71).

The dopamine transporter (DAT) mediates reuptake of the neurotransmitter dopamine from the synaptic cleft back into the presynaptic neuron and thereby terminates dopaminergic signaling. DAT is part of the monoamine transporter family (MAT) and the larger Neurotransmitter Sodium Symporter (NSS) family^1^. The MAT family also includes the serotonin and norepinephrine transporters (SERT and NET), which are all located in the presynaptic plasma membrane of neurons^2–4^. The MATs have a conserved architecture consisting of 12 transmembrane helices (TM) (Fig. 1a) with a central substrate binding site^5,6^. For neurotransmitter translocation the MATs use the transmembrane sodium gradient as a driving force^7^. Dysregulation of the MATs has been linked to several major diseases such as depression^8^, Parkinson disease^9^ and attention deficit hyperactive disorder^10^. The transporters have also been implicated in drug addiction^11,12^. The means of regulation and supramolecular organization of the MATs in the plasma membrane is therefore of great interest for application in drug development^13^.

Membrane protein oligomerization is increasingly observed to play an important role in protein function and regulation, and the MATs are no exception^14,15^. The MATs have been experimentally shown to form homooligomers^16,17^, and specifically in the synaptic plasma membrane several oligomeric states have been detected^18^. MAT oligomerization is presumed necessary for endoplasmic reticulum export^16^ and for DAT it has been suggested that the oligomeric state can have a cooperative effect on inhibitor binding and substrate transport^19^. From cysteine cross-linking experiments there is evidence suggesting that SERT and DAT form a homotetramer presumably consisting of a dimer of dimers^20,21^, but from more recent single-molecule experiments other oligomeric states have also been observed for hSERT^18^, and for hDAT only the monomeric and dimeric states have been detected in the plasma membrane^20^.

Currently, no consensus exists with regard to the oligomeric interfaces present within and across MATs in the plasma membrane. Symmetric interfaces involving either TM4, TM6, or TM2 have been suggested for DAT from cysteine cross-linking and mutagenesis experiments^17,20,22,23^. Computational protein–protein docking studies indicate that TM2, TM6, and TM11 in union constitute a symmetrical dimeric interface in hDAT^23^. For SERT, FRET experiments of the TM fragments TM1-2 and TM11-12 point to these pairs forming a symmetrical dimer interaction site^24^. Crystal structures of a prokaryotic NSS member, the leucine transporter (LeuT), presents a symmetrical dimer consisting of an interface involving TM9, TM12, and extracellular loop 2 (EL2)^25^. In summary, a wide range of different interfaces have been detected and proposed to be important for hDAT and hSERT oligomerization. Collectively, the studies do not point to a unique single dimer model for all MATs, rather several interfaces for the same transporter are likely to exist.

Recently, we successfully presented data indicating that a likely dimer candidate for hSERT in a 1-palmitoyl-2-oleoyl-*sn*-glycero-3-phosphocholine (POPC) membrane consists of a symmetrical TM12/TM12 interface (the naming convention applied throughout the manuscript for indicating which helices are found at the dimer interface. TM helices contacting from the same protomer are separated by commas, while a slash is used to indicate the second protomer’s interacting helices)^14^. The TM12/TM12 interface is in partial agreement with previous FRET studies^24^ and the interface observed in crystal structure of the prokaryotic NSS member, the leucine transporter (LeuT)^25^. We employed a combination of multiple self-assembly simulations followed by umbrella sampling replica exchange molecular dynamics (US-REMD) simulations on a subset of highly populated hSERT dimer conformations to determine potential of mean force (PMF) free energy profiles of the interfaces^14^. From the same work, we further show that the lipid composition of the embedding membrane largely affected the strength of the TM12/TM12 interface. Lipids bound to the surface of membrane proteins in general, could therefore either stabilize or destabilize certain dimeric or higher order oligomeric states^26,27^. In the case of DAT, a palmitoyl (palm) chain is reversibly attached at TM12 on Cys581 (Fig. 1a) and has been shown to regulate DAT surface expression and turnover^28,29^. Palm is a post-translational modification involving the thioesterification of palmitate, a 16-carbon fatty acid, to a cysteine residue. For integral membrane proteins, this modification is reversible and regulates a variety of properties including functional activity, trafficking, turnover, membrane raft targeting, and cholesterol binding^30–34^. Due to the location of the palm group on TM12, which has been shown from both LeuT crystal structures and hSERT self-assembly simulations to be involved in the dimer interface, we speculated that the palm group may have a regulatory mechanism on hDAT dimer formation, similar to what has been observed for some GPCRs^35^. In this contribution, we employ our previous approach used for hSERT to investigate the impact of S-palmitoylation on hDAT oligomeric behavior^14,36^. We identify several interfaces and show that the stability of dimer interfaces involving TM12 changes when TM12 is palmitoylated. We also compare the LeuT dimer from crystal structures to those obtained from self-assembly simulations and find that similar helices are involved at the interface for LeuT, hDAT and hSERT. Collectively, we propose that the MATs share a common dimer pattern, which mainly involves the helices TM9, TM11, and TM12, and that S-palmitoylation may act as a functional switch.

## Results

### Palmitoylation of hDAT barely affects the interhelical contacts formed in self-assembly simulations

In order to study which helices are most prevalent at the oligomeric interfaces of hDAT and how these are affected by hDAT being S-palmitoylated (hDAT-palm) on TM12, we performed 10 self-assembly simulations each lasting 30 µs and containing 16 hDAT or 16 hDAT-palm proteins embedded in a pure POPC membrane bilayer (Fig. 1b). Visual inspection of the last frames of the different repeat simulations indicates that both hDAT and hDAT-palm form dimers and higher order oligomers on a µs timescale (Fig. 1a and Supplementary Fig. S1 for a full overview)^14^.

To investigate which helices are most prevalent at the interface, interhelical contacts were quantified between protein pairs. Interhelical contacts were calculated by counting the number of times a helix from one protomer is in contact with another helix of another protomer, but not considering the palmitoyl group. The interhelical contacts were summed across all frames and repeat simulations to consider all possible dimer pairs. Illustrated in Fig. 1c (central panel) is the proportion of times each helix (TM1 to TM12) is found to form a contact with any other helix in another protein. It is observed that in the simulations of hDAT with and without the S-palmitoylation, the helices that frequently form contacts are similar in the two systems (Fig. 1c, middle panel). Specifically, helix contacts involving TM9, TM11, and TM12 and to some extent TM4-6 are predominant in both systems. We then assessed whether the helices frequently in contact are correlated with being more accessible to the surrounding lipid environment by computing the lipid accessible surface area (LASA) of the 12 different helices using a spherical probe with a radius of 0.26 nm (Fig. 1c, top panels). From the LASA, it is detected that the helices, TM4, TM11, and TM12, which are frequently present at the dimer interface, are also the most exposed to the lipid environment of all the TM helices (Fig. 1c, top panels). In contrast, TM9, which is the most contact-forming helix, is only marginally exposed. It is also notable that TM7, which is more exposed than TM9, forms few contacts. These observations suggest that the exposure of a TM is not the main factor determining the formation of contacts.

Illustrated in the bottom panels of Fig. 1c are 2D contact maps revealing interhelical pairs in the simulations that most frequently form contacts. Interhelical contact matrices share a comparable pattern in the two systems (Fig. 1c, bottom panels) indicating that similar helices are found at the dimer interface in the two systems. However in the simulations where hDAT is palmitoylated, TM12 of one protomer is in less contact with TM12 of another protomer compared to simulations without palmitoylation. Otherwise the two systems agree on TM12 of one protomer being frequently in contact with TM4-5 and TM9 of the other protomer. TM9 being frequently in contact with TM4, TM9, TM11, and TM12, and TM11 being frequently in contact with TM4 and TM11. The relative distributions of the helix pairs are, however, slightly different in the two systems.

Two motifs have been suggested to promote integral protein oligomerization: a leucine zipper motif and a GXXXG motif ^37,38^. The MATs contain a conserved GXXXG motif in both TM6 and EL3, and hDAT has an additional leucine zipper in TM9 (the locations of the motifs are highlighted in Supplementary Fig. 2). However, the two conserved GXXXG motifs are buried inside the protein and cannot partake in a dimer interface. The leucine zipper motif in TM9 of hDAT is exposed to the membrane milieu and the majority of leucine residues can readily form contacts to another protomer (see Supplementary Fig. 3). It is therefore noteworthy that TM9/TM9 contacts are frequently observed for both the hDAT and hDAT-palm system.

**Figure 2:**
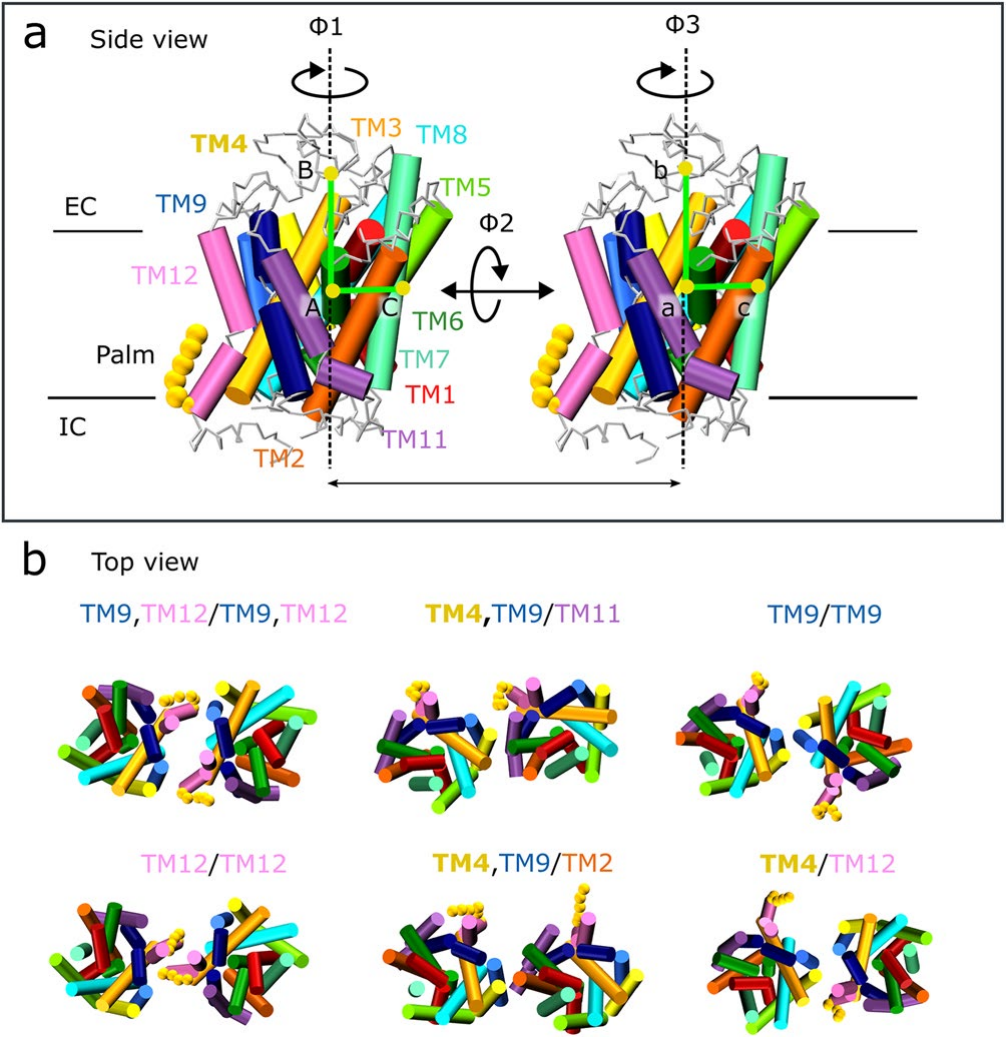
Dimer cluster comparison between the two systems, hDAT and hDAT-palm. Depicted in (**a**) is a transmembrane (TM) overview of hDAT shown from the side with respect to the membrane normal. The helices are individually color-coded using the same coloring scheme as applied throughout the article. Highlighted in bold spheres is the palmitoyl group (palm) covalently bound to TM12. The two angles, ϕ1 and ϕ3, are used for describing the individual protomer’s relative orientation. Depicted in (**b**) are the common top clusters observed in both the hDAT and hDAT-palm simulations, with the exception of the TM12/TM12 dimer, which is only detected 0.1% of the time in the hDAT-palm simulations. For a full view of the representative dimers of all top clusters of the hDAT and hDAT-palm system see Supplementary Fig. S5-6.

**Figure 3:**
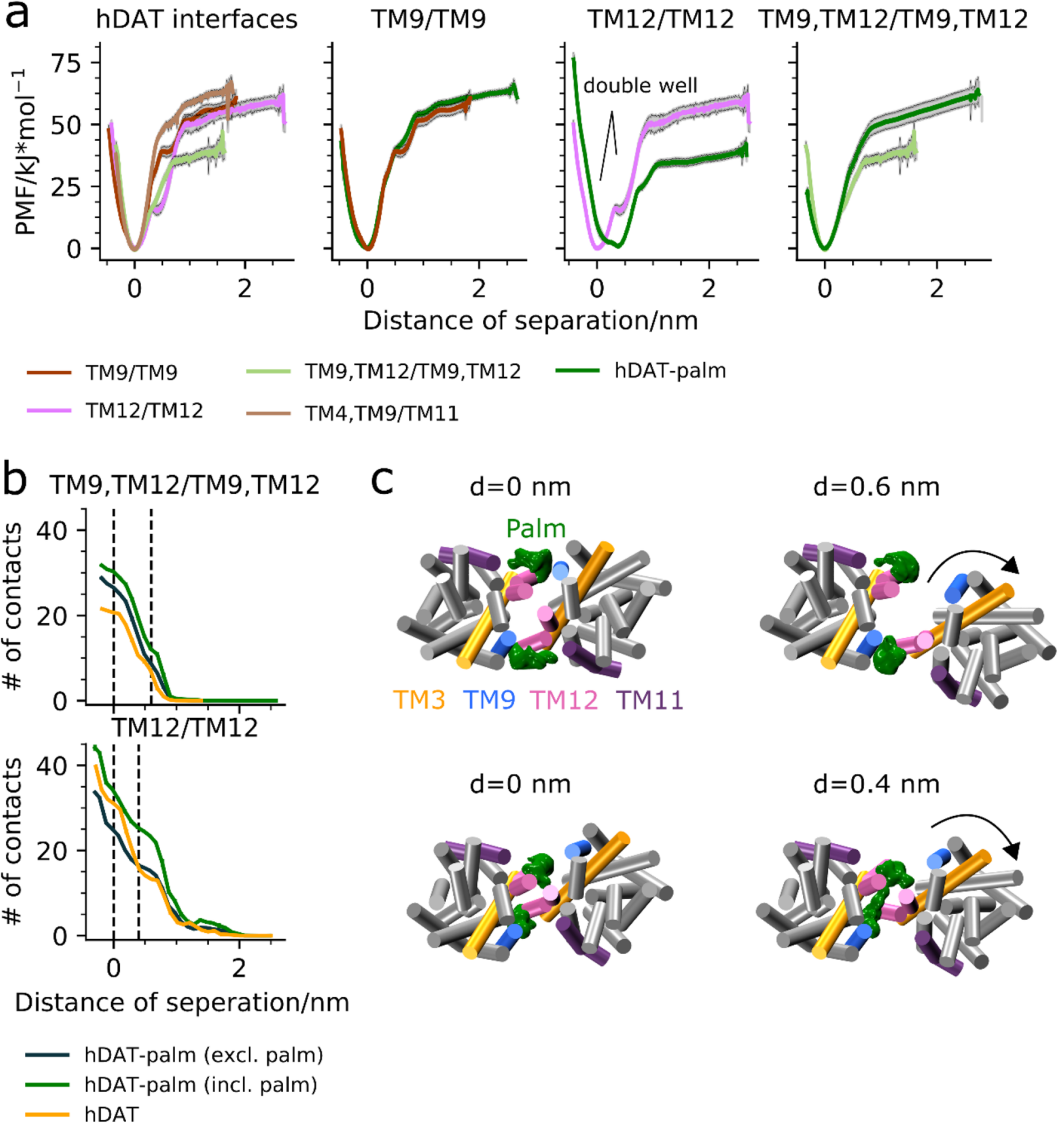
Potential of mean force energy profiles and contact analysis of hDAT and hDAT-palm interfaces. Illustrated in the first panel in (**a**) are all the PMFs of the different hDAT interfaces: TM9/TM9 (brown), TM9,TM12/TM9,TM12 (light green), TM12,TM12 (pink), and TM4, TM9/TM11 (light brown). Shown in the subsequent plots are PMF comparisons of the interfaces TM9/TM9, TM9,TM12/TM9,TM12, and TM12/TM12 between hDAT and hDAT-palm (green). Shown in (**b**) are the average number of residue contacts calculated within 1 Å distance bins along hDAT dimer separation relative to the distance at energy minimum as detected from the PMF energy profiles. The number of residue contacts for the two systems, hDAT and hDAT-palm, at the two interfaces, TM12/TM12 and TM9, TM12/TM9, TM12, were monitored. For both interfaces the Cys581 residue where palm is attached is either included (incl.) or excluded (excl.) from the contacts analysis. A contact was counted when the distance between two residue pairs was below 7 Å. Each contact was unbiased. Error bars have been included by performing bootstrapping on the data using 10 iterations, although they are barely visible. Shown in (**c**) are representative dimer conformations of hDAT-palm at different separation distances for the TM9, TM12/TM9, TM12 interface (top) and the TM12/TM12 interface (bottom). Highlighted are helices TM3, TM9, TM11 and TM12. The green mesh corresponds to the palm occupancies calculated using the Volmap plugin in VMD for the dimer conformations found around the given separation distance. For the Volmap calculations the size of the system beads were set to 2.6 Å and the “occupancy” setting was selected. The arrows indicate the movement of the proteins with respect to each other.

### hDAT with and without a palm group results in similar dimers

To characterize further the dimer conformations formed in the self-assembly simulations of hDAT and palmitoylated hDAT, we performed a cluster analysis of the proteins in contact (see Methods for further details and Supplementary Fig. S4). In agreement with the contact analysis, the cluster analysis shows that similar protein dimers exist for the hDAT and hDAT-palm systems with slightly differing relative populations (Fig. 2 and Table 1). The predominant dimer conformations found in both systems consist of the following interfaces evaluated by visual inspection: TM9/TM9, TM4,TM9/TM11, TM4,TM9/TM2, TM9,TM12/TM9,TM12 and finally TM12/TM4 (Fig. 2b). The most populated clusters for both hDAT and hDAT-palm dimers primarily involve TM9 and/or TM11 helices at the interface (see Supplementary Fig. S5-6). The TM12/TM12 interface of the palmitoylated hDAT is also presented in Fig. 2b, although it is only detected 0.1% of the time during simulations according to the cluster analysis. The TM12/TM12 interface is included and studied further in a later section because I) it resembles the hSERT TM12/TM12 interface^14^ and II) it involves TM12 on which palm is attached. Based on the cluster analysis, TM12 is involved more frequently at dimer interfaces found in the hDAT system compared to the hDAT-palm system. The reason for this, is due to the presence of the long flexible palm group that prevents the formation of interprotein contacts. Thus, only short-lived contacts can form at much longer distances than the center-of-mass (COM) cut-off distance applied for defining a dimer pair used in the cluster analysis (see Methods and “TM12/TM12 dynamics” sections for further details).

**Table 1:** hDAT and hDAT-palm cluster properties. The cluster number and cluster size for the different dimers is given for the system with ( +) and the system without (−) palm. The dimers relative orientation is given by the ϕ1 and ϕ3 angle (see Fig. 2a) and the average ΔLASA for the different dimer interfaces is listed. Notice that some clusters come in pairs e.g. cluster 7,8 due to symmetry considerations, which is elaborated upon in the Methods section under clustering.

PALM interface	Cluster number		Cluster size/%		Φ1, Φ3/°		ΔLASA/nm^2^	
	−	+	−	+	−	+	−	+
TM9,TM12/TM9,TM12	1, 2	10	13.4	3.0	− 31, − 24	− 34, − 28	16.0	17.6
TM4,TM9/TM11	3, 4	2, 3	10.0	13.0	30, − 127	26, − 123	15.5	16.3
TM9/TM9	6	1	4.3	11.1	3, 0	0, 0	20.8	20.7
TM12/TM12	9	53	3.0	0.1	− 46, − 47	− 41, − 48	12.5	15.2
TM4,TM9/TM2	7, 8	8, 9	7.0	6.1	177, 15	165, 4	18.7	18.7
TM4/TM12	10, 11	11, 12	5.5	4.5	34, − 47	18, − 29	12.5	12.0

For describing the relative orientation of the individual protomers with respect to each other in the different dimer conformations two dihedral angles were used^36^, ϕ1 and ϕ3 (Fig. 2a) as defined in the Virtual Bond Algorithm^39^. The values of ϕ1 and ϕ3 for the different dimer conformations are given in Table 1. In principle, a total of three dihedral angles (see Fig. 2a) are necessary for thoroughly understanding two rigid bodies relative orientation, however, it is observed in our system that the ϕ2 angle does not vary more than ± 25°, due to the proteins being embedded in a membrane. The ϕ2 angle is therefore not considered further. When the ϕ1 and ϕ3 values are similar, the dimers can be regarded as symmetrical with respect to a C2 axis perpendicular to the membrane plane e.g. the dimers TM9/TM9, TM12/TM12, and TM9,TM12/TM9,TM12. Whereas when the ϕ1 and ϕ3 values are dissimilar the dimers are anti-symmetric e.g. the dimers TM4,TM9/TM11 and TM4,TM9/TM2. In addition, it can also be observed which clusters are closely related e.g. cluster 1,2 and 9 for hDAT (see Table 1 and Supplementary Fig. S7).

The average degree of surface area occlusion of the dimer interfaces found in the different clusters varies between 12 and 20.7 nm^2^ and was computed by subtracting the LASA of the full dimer complex from the LASA of the two individual monomers. The TM9/TM9 interface is the highest excluded lipid exposed area for both the hDAT and hDAT-palm interfaces. However, in agreement with the contact analysis, there is no clear pattern between the dimers observed to be among the top clusters and the degree in which their interface is lipid exposed (Table 1).

A limitation of the MARTINI force field is the inherent stickiness of the model^40^, despite this, however, we do see protein unbinding events. Moreover, the protein surface probed is similar when comparing the two sets of five repeat simulations (see Supplementary Fig. S8), suggesting a reasonably converged surface exploration. To evaluate in a more quantitative manner the strength of the highly populated dimer interfaces detected in the cluster analysis, we computed the PMF along protomer separation using US-REMD simulations.

### Palmitoylation of TM12 changes the energetics of dimer interfaces involving TM12

In the following section, we compare the interface’s strength of a subset of different dimer conformations by generating their PMF energy profiles from US-REMD simulations. The hDAT dimer conformations selected to be evaluated through PMF energy profiles consisted of TM9,TM12/TM9,TM12 (cluster 1), TM4,TM9/TM11 (cluster 3,4), TM9/TM9 (cluster 6), and TM12/TM12 (cluster 9). These interfaces are found among the most populated clusters of the hDAT self-assembly simulations (Fig. 2b). Similarly, the hDAT-palm interfaces TM9,TM12/TM9,TM12 (cluster 10), TM12/TM12 (cluster 53), and TM9/TM9 (cluster 1) were also evaluated, to further understand the effect of palm on the dimer interface strength. The hDAT-palm TM9/TM9 interface was primarily used as a control, since palm is not involved at this interface. The energetics is therefore expected to be unchanged between the hDAT and the palmitoylated hDAT system. The protocol used for generating the PMF’s has previously been applied by Periole et al. on hSERT and on GPCRs^36^ and is briefly described in the Methods section ^14^. It should be highlighted that the aim was to rank the conformations and not to establish absolute free energies.

The PMFs for the different hDAT interfaces are presented in the first panel in Fig. 3a (see Supplementary Table S1-2 for further details concerning the US-REMD settings). The three hDAT dimer interfaces, TM12/TM12, TM4,TM9/TM11, and TM9/TM9, have a similar value of free energy of dissociation of 55 ± 10 kJ/mol. The TM9,TM12/TM9,TM12 interface is weaker with a dissociation free energy of ~ 40 kJ/mol. Interestingly, the TM12/TM12 interface seems to have a second minimum (double well) at a separation distance of ~ 0.4 nm (clear in panel 3 in Fig. 3a).

When comparing the PMF profiles of the TM9/TM9 interface between hDAT and hDAT-palm (Fig. 3a, second panel), it is observed that the profiles are very similar, emphasizing that the sampling of the dimer conformations along the reaction coordinate is sufficient. This similarly demonstrates that Cys581 S-palmitoylation of TM12 has no global effect on dimer binding. In contrast, the PMF profiles of the interfaces involving TM12 change with hDAT palmitoylation. The TM9,TM12/TM9,TM12 interface is relatively stabilized upon TM12 S-palmitoylation, whereas the TM12/TM12 interface is relatively destabilized. Furthermore, the global minimum for the hDAT-palm TM12/TM12 interface is solely found in the second long distance minimum observed for the hDAT TM12/TM12 interface (Fig. 3a, third panel).

To further understand the mechanism by which S-palmitoylation affects the energetics of the TM12/TM12 and TM9,TM12/TM9,TM12 interfaces, we determined the average number of (residue-based) contacts formed in the US-REMD simulations for both the hDAT and hDAT-palm systems (Fig. 3b). We assessed palm’s involvement at the two interfaces by both including and excluding the group from the contact analysis. As expected, the number of contacts increases as the distance between the proteins decreases, and palm adds a significant contribution to the total number of contacts formed at both interfaces (Fig. 3b). It is observed at the free energy minimum of the two interfaces (d = 0 nm) that palm increases the number of non-palm interprotein contacts for the TM9,TM12/TM9,TM12 interface, but decreases the number of non-palm interprotein contacts for the TM12/TM12 interface (Fig. 3b). Thus, the strength of the TM12/TM12 and the TM9,TM12/TM9,TM12 interfaces is correlated with the total number of non-palm protein–protein contacts.

The number of non-palm interprotein contacts in the hDAT-palm TM9,TM12/TM9,TM12 interface is higher than for the corresponding hDAT interface, indicating that the two proteins can form additional contacts when palm is present, in contrast to what one might expect (Fig. 3b, top panel). We computed the average ϕ1 and ϕ3 angle values for all dimers found in the TM9,TM12/TM9,TM12 clusters (cluster 1 vs. cluster 10, see Table 1) for the hDAT and hDAT-palm systems, respectively, and detected a 5.4° average difference. This slight difference in relative orientation between the hDAT and the hDAT-palm TM9,TM12/TM9,TM12 dimer interface could explain why additional contacts can form in the hDAT-palm system. In fact, when visualizing the palm 3D occupancy maps around the energy minimum, we observe that palm is located in the nook formed between TM9 and TM12 (Fig. 3c, top panel). Therefore, palm does not seem to disrupt the formation of non-palm inter-protein contacts, rather it could result in better protein–protein packing possibly due to this slight dimer reorientation. For both the hDAT and the hDAT-palm systems a small shoulder is observed in the contact analysis at d =  ~ 0.6 nm (Fig. 3a, fourth panel), which corresponds to a reorientation of the TM9,TM12/TM9,TM12 interface resulting in loss of contacts to a single TM9/TM12 pair (Fig. 3c, top panel).

Looking instead at the contact analysis for the hDAT-palm TM12/TM12 interface, a decrease in non-palm interprotein contacts at the free energy minimum (d = 0 nm) relative to the hDAT system is observed (Fig. 3b, bottom panel). This decrease may easily be rationalized by palm getting squeezed between the TM12 helix of one protomer and the protein of the other, thereby preventing certain non-palm interprotein contacts from forming (Fig. 3c, bottom panel). At the distance of the second free energy minimum of the hDAT TM12/TM12 interface (d =  ~ 0.4 nm), the number of protein contacts in the hDAT and hDAT-palm (excluding palm) system are similar. However, when palm is included in the analysis, a higher number of contacts is detected for the hDAT-palm system (Fig. 3b). At the d =  ~ 0.4 nm distance, palm seems to stabilize the TM12/TM12 interface and it is observed that palm-palm contacts can form, by bridging across the TM12/TM12 interface (Fig. 3c, bottom panel).

To summarize, the PMFs show that palm changes the energetics of the interfaces involving TM12 and the contact analysis along the interface separation distance shows that this is caused by palm being present at the interface and thereby i) providing, in all cases, additional contacts to form ii) increasing existing protein–protein contacts (in the TM9,TM12/TM9,TM12 interface) or iii) preventing protein–protein contacts to form (in the TM12/TM12 interface).

### The dynamics of the TM9/TM12 interface and the effect of palmitoylation

**Figure 4:**
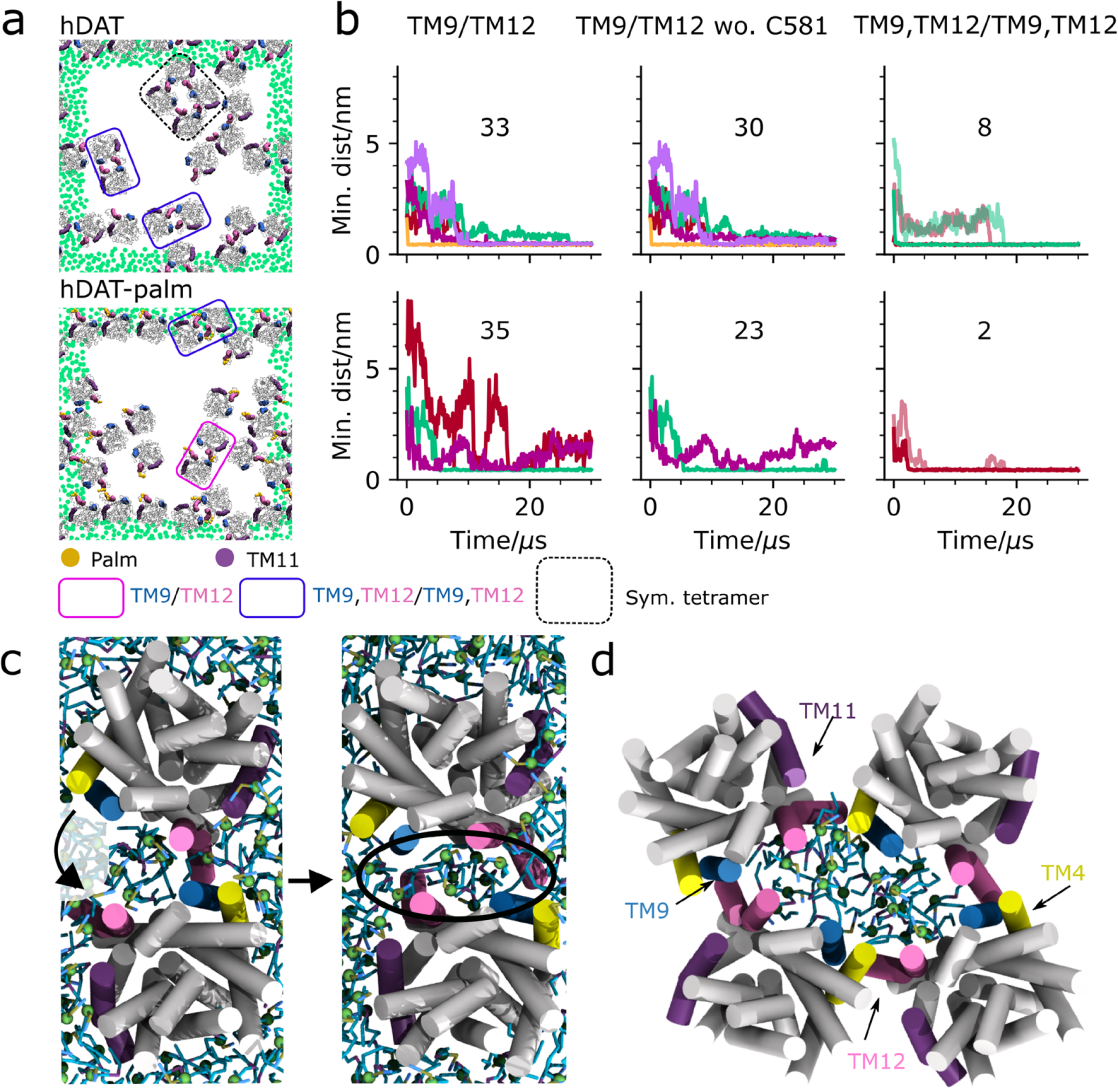
Dynamics of the TM9/TM12 interface. In (**a**) the last frame of a single representative repeat simulation is shown for hDAT (MD1, top) and hDAT-palm (MD4, bottom). The last frames for all repeat simulations are supplied in Supplementary Fig S1. Within the pink boxes are TM9/TM12 interfaces and illustrated in the purple box is a symmetrical interface involving both TM9 and TM12 helices, dubbed TM9, TM12/TM9, TM12. Highlighted in the black dotted box is a symmetrical tetramer. The green dots correspond to the GL1 bead in POPC and are depicted for the nearest periodic images to the system. For the single simulation box the dots have been omitted. The helices TM9, TM11, and TM12 are highlighted in blue, purple, and mauve, respectively. Illustrated in (**b**) is the formation of single TM9/TM12 contacts both considering and not considering Cys581 (wo. Cys581) on which palm is attached in the analysis (the first and second column, respectively). Double TM9,TM12/TM9,TM12 contacts are also monitored for the same representative repeat simulations as depicted in (**a**) for hDAT (top) and hDAT-palm (bottom)(third column). Each color in the same plot represents a different dimer pair and the total number of dimers across all repeat simulations that form the given interface are noted in the plots. The TM9, TM12/TM9, TM12 interface is further subdivided into two similar color shades representing the minimum distance for each TM9/TM12 helix pair located in the same dimer. The contact plots for the different interfaces across all repeat simulations are supplied in Supplementary Fig. S10-14. In (**c**) the formation of the symmetrical TM9, TM12/TM9, TM12 interface is shown. It is observed that POPC lipids are associated with the interface. Illustrated in (**d**) is a close-up of the symmetrical tetramer.

Visual inspection of the last frames of the 10 different hDAT and hDAT-palm repeat simulations shows that both single TM9/TM12 interfaces and double TM9,TM12/TM9,TM12 interfaces are formed, as highlighted in Fig. 4a by pink and purple boxes, respectively (see Supplementary Fig. S1 for a full overview). To evaluate the dynamics of the TM9/TM12 interfaces we monitored the formation of single TM9/TM12 dimer interfaces as a function of simulation time for all TM9/TM12 pairs that were in contact continuously for more than 500 ns during the course of the simulation. Figure 4b illustrates that TM9/TM12 dimers are formed 33 times in the hDAT system and 35 times in hDAT-palm system. If Cys581, on which palm is bound is excluded from the contact analysis (Fig. 4b, middle column) then the number of dimers forming a TM9/TM12 contact in the hDAT-palm system decrease from 35 to 23. In the hDAT system, this change is less prominent, 33 vs. 30, respectively. Collectively, the total number of frames forming TM9/TM12 contacts drops by 0.1% in the hDAT system and 47% in the hDAT-palm system when Cys581 is excluded from the contact analysis. These results indicate that the flexible palm group helps TM9/TM12 contacts to form at longer distances, but that palm at these longer distances is the primary group forming interprotein contacts.

While monitoring the formation of the TM9,TM12/TM9,TM12 interface over time, it was revealed that first single TM9/TM12 contacts form followed by the remaining TM9/TM12 helices coming together (Fig. 4b,c). This dynamical behavior is similar to that observed in the US-REMD simulations (Fig. 3c, top panel). In both systems the formation of the double TM9,TM12/TM9,TM12 interface takes multiple µs to form after the single TM9/TM12 helices have come together, and the closure of the interface results in the encapsulation of POPC lipids in the space between the proteins (highlighted by a black ellipsoid in Fig. 4c). The number of POPC lipids that get encapsulated by the TM9,TM12/TM9,TM12 dimer are approximately five and eight for the hDAT-palm and hDAT system, respectively.

The TM9,TM12/TM9,TM12 hDAT dimer was converted to atomistic resolution and simulated for 530 ns. During the simulation we observe a slight reorientation of the proteins in which contacts to a single TM9/TM12 pair is lost (see Supplementary Fig. S9). This loss in contacts allows for POPC lipids from the surrounding milieu to enter the otherwise enclosed space. Once again illustrating that the TM9,TM12/TM9,TM12 interface is very dynamic. On the other hand, it could also be indicative of the interface not being stable and thus not necessarily biologically relevant. However, more likely, the dimer rearrangement is caused by the difficulty in equilibrating the interfacial lipids.

In the self-assembly simulations a total of eight dimers form double TM9,TM12/TM9,TM12 interfaces in the hDAT system and two in the hDAT-palm system (Fig. 4b, see Supplementary Fig. S10 and Fig S12 for a full overview). Excluding Cys581 from the contact calculations in Fig. 4b does not change the number of dimers forming symmetrical TM9,TM12/TM9,TM12 dimers in the two systems. Moreover, according to US-REMD simulations, once non-palm interprotein contacts form, the interface becomes more stable in the presence of palm (Fig. 3b). Nonetheless, the TM9,TM12/TM9,TM12 interface is formed more rarely in the hDAT-palm self-assembly simulations, possibly due to the exclusion of POPC molecules from the interface space having a higher energy barrier in the presence of palm and/or palm’s increased flexibility requires a higher amount of sampling to thoroughly probe the TM9,TM12/TM9,TM12 interface.

Of potential interest, is the observation that a symmetrical tetramer in two hDAT repeat simulations forms consisting of TM9/TM12 contacts in addition to TM4/TM12 helix contacts (Fig. 4d). A similar dimer of dimers construction has previously been suggested for hDAT^20^.

### Dynamics of the TM12/TM12 interface and the effect of palm

**Figure 5:**
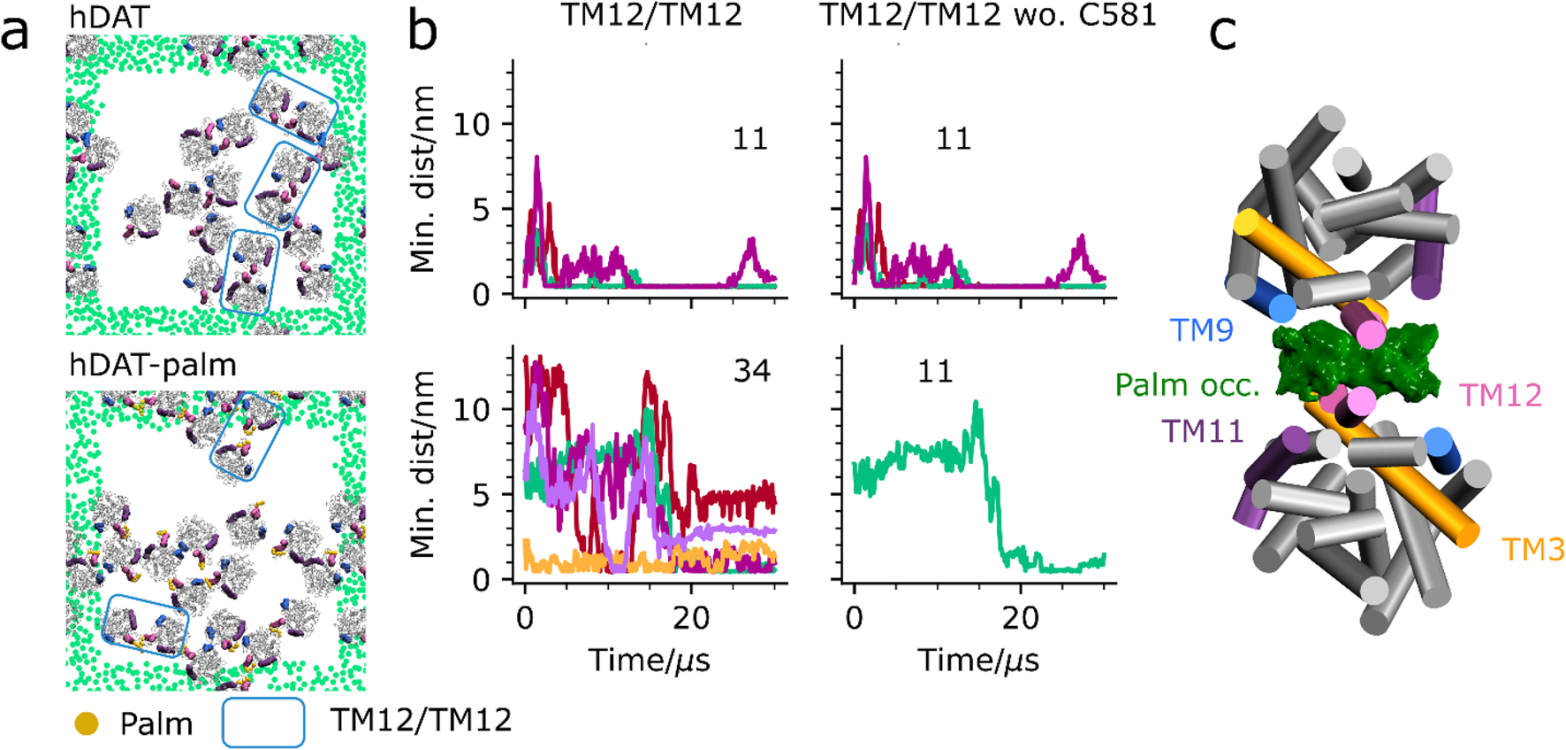
Dynamics of the TM12/TM12 interface. In (**a**) the last frame of a single representative repeat simulation is shown for hDAT (MD2, top) and hDAT-palm (MD3, bottom). The last frames for all repeat simulations are supplied in Supplementary Fig. S1. Similar interfaces are observed across the two systems involving TM12 on which palm (orange) is attached. Within the blue box a TM12/TM12 interface is observed. The green dots correspond to the GL1 bead in POPC and are shown for the nearest periodic images to the system. For the single simulation box the dots have been omitted. The helices TM9, TM11, and TM12 are highlighted in blue, purple and mauve, respectively. In (**b**) the formation of single TM12/TM12 contacts both considering and not considering Cys581 (wo. Cys581) on which palm is attached in the analysis. The TM12/TM12 pairs that were monitored were evaluated as being in contact continuously for 500 ns during the course of the simulation. A contact was defined when the minimum distance between TM12 helices was below 7 Å. The contact plots for the different interfaces across all repeat simulations are supplied in Supplementary Fig. S15-17. Shown in (**c**) is the palm 3D occupancy computed using the Volmap plugin in VMD for all dimers captured in the TM12/TM12 hDAT-palm cluster (cluster 53 in Table 1).

The TM12/TM12 interface was previously found to be the most energetically favorable for hSERT and was also the most frequently formed in hSERT self-assembly simulations (18%)^14^ and has been partly observed in X-ray structures^5^. The TM12/TM12 interface is similarly formed in the hDAT and hDAT-palm self-assembly simulations according to the per helix contact analysis, cluster analysis and visually based on the last frame of the different repeat simulations (Fig. 1b, Fig. 2b and Fig. 5a, respectively). It is, however, not as populated as other interfaces (3.0% and 0.1% for hDAT and hDAT-palm, respectively), nor markedly more stable based on the PMF energy profiles (Fig. 3a). The number of TM12/TM12 pairs that form contacts lasting more than 500 ns in the hDAT simulations both considering and not considering a contact formed by Cys581 is unchanged (11 in Fig. 5b). In the hDAT-palm simulations, a higher number of TM12/TM12 dimers form compared to in the hDAT simulations, 34 vs. 11, respectively. However, these dimers are mostly engaged by palm itself and are therefore short-lived and unstable. Only 11 out of 34 dimers remain if palm (Cys581) is excluded from the contact analysis. Corresponding to a 74% drop in the relative number of frames forming TM12/TM12 contacts (Fig. 5b). By computing the 3D occupancy of palm using the TM12/TM12 dimer conformations captured in the cluster analysis, it is clearly observed that palm is involved at the interface (Fig. 5c). Taken together, this data illustrates that the palm group promotes dimer contacts at longer distances which are not stabilized by protein–protein contacts and that in the presence of palm, the TM12/TM12 interface becomes less stable. From the cluster analysis only 0.1% TM12/TM12 hDAT-palm dimers are observed, which further illustrates that the TM12/TM12 interface is difficult to capture using the clustering method, due to palm increasing the interprotein distance and the relative protein orientations that lead to TM12/TM12 contacts.

### Comparison of the LeuT interface to the hDAT TM9,TM12/TM9,TM12 and hSERT TM12/TM12 interface

**Figure 6:**
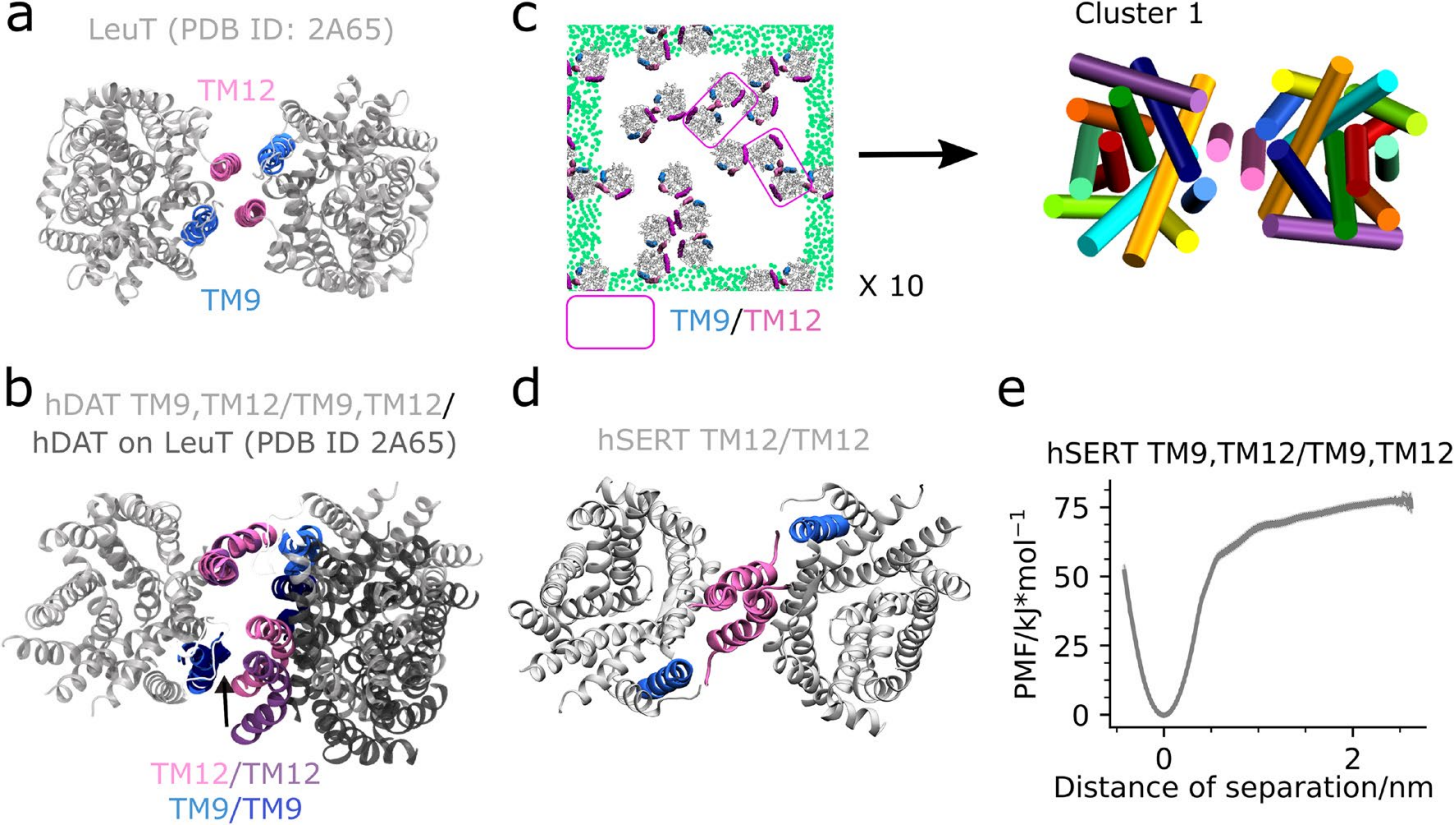
LeuT and MATs have a similar TM12/TM12 and TM12,TM9/TM12,TM9 interface. Shown in (**a**) is a top view of the LeuT crystal structure dimer (PDB ID: 2A65) with helices TM9 and TM12 highlighted. In (**b**) the central hDAT dimer structure from cluster 1 (see Fig. 2b) is superimposed on hDAT monomers aligned to the LeuT dimer. Depicted in (**c**) is a representative last frame of the 10 LeuT self-assembly repeat simulations (MD1). See Supplementary Fig. S18 for a full overview. Each system contains 16 LeuT (PDB ID: 2A65) proteins equally spaced but randomly orientated in a pure POPE membrane and has been simulated for 30 µs. The helices TM9, TM11, and TM12 are highlighted in blue, purple, and mauve, respectively. Emphasized by pink boxes are dimers containing TM12/TM12 and to some extent TM9 contacts. The green dots correspond to the GL1 bead in POPE and are shown for the nearest periodic image to the system. For the central simulation box the dots have been omitted. To the right in (**c**) is a top view with respect to the membrane normal of the central dimer conformation observed in cluster 1. Notice that the dimer achieved from our simulations has an interface consisting of TM12/TM12, TM9 contacts, due to a slight change in the two proteins relative orientation. This change in orientation relative to the LeuT crystal structure dimer results in a loss of contacts to one of the TM9 helices. Represented in (**d**) is the stable hSERT TM12/TM12 dimer previously detected^14^. Finally, in (**e**) is the PMF of the hSERT TM9,TM12/TM9,TM12 interface.

The TM9,TM12/TM9,TM12 dimer interface observed to be among the top clusters for both hDAT and hDAT-palm (Fig. 2b) is similar to that present in crystal structures of a MAT bacterial homologue, LeuT (Fig. 6a). However, the hDAT TM9,TM12/TM9,TM12 dimer differs in the relative orientation of the TM12 and TM9 helices in addition to TM12 being kinked in hDAT and being straight in LeuT (Fig. 6b). When aligning two hDAT monomers on the LeuT crystal structure (PDB ID: 2A65)^25^ dimer using Pymol’s align command the symmetrical dimer achieved (hDAT-on-LeuT) is surprisingly similar to the central dimer conformation of cluster 1 from the hDAT self-assembly simulations (Fig. 2b). The RMSD between the two dimers Cα atoms is only 3 Å. The main difference is that TM12 is shifted slightly closer to TM9 in the dimer conformation observed in our simulations as compared to the hDAT-on-LeuT dimer (Fig. 6b).

LeuT has repeatedly been observed to crystallize as a TM9,TM12/TM9,TM12 dimer^25^. However, this does not necessarily make it biologically relevant, and the dimer may simply be influenced by the crystallization environment. Using MS/MS techniques it has been confirmed that LeuT forms a dimer in a lipid environment^26^, but it is yet unclear whether this dimer consists of the TM9,TM12/TM9,TM12 interface observed in crystal structures. To evaluate whether LeuT forms a similar interface during self-assembly simulations, we ran 10 repeat simulations of LeuT (PDB ID: 2A65) randomly orientated in a POPE membrane and subsequently clustered the generated dimers. Strikingly, the dimer conformation observed in the top cluster consists of TM12/TM12 contacts with some involvement of TM9 and occurs 12% of the time (see Fig. 6c and Supplementary Fig. S18-20 for additional information on the clustering of the LeuT dimers). Using the ϕ1 and ϕ3 dihedral angles for describing the relative orientation of the LeuT protomers in the different dimers observed, we found that these angles were − 13.2° and − 12.2° for the central dimer conformation of the most populated cluster and − 7.3° and − 7.3° for the LeuT crystal structure dimer (PDB ID: 2A65), respectively (see Supplementary Fig. S21). From visual inspection of the central LeuT self-assembly dimer of cluster 1 aligned to the Cα atoms of the crystal structure of LeuT (RMSD value of 4.4 Å), the slight change in the two dihedral angles reflects the LeuT dimer having a TM9,TM12/TM12 interface rather than a symmetrical TM9,TM12/TM9,TM12 interface in self-assembly simulations. Interestingly, this denotes to a mix of the hDAT TM9,TM12/TM9,TM12 interface and the hDAT and hSERT TM12/TM12 interface (Fig. 6d). In fact, the MAT TM12/TM12 and TM9,TM12/TM9,TM12 interfaces are closely related when comparing their ϕ1 and ϕ3 angle values, which do not differ more than ~ 10° (Table 1).

Due to the strength and prevalence of the TM9,TM12/TM9,TM12 interface we revisited our hSERT self-assembly data and did in fact observe a single TM9,TM12/TM9,TM12 interface being formed. Using this dimer, we calculated its PMF and found that the TM9,TM12/TM9,TM12 interface is in fact also stable for hSERT and even stronger than the TM12/TM12 interface, ~ 75 kJ/mol vs. ~ 55 kJ/mol, respectively^14^ (Fig. 6e). Collectively, the high similarity between the populated LeuT self-assembly dimer and the crystal structure dimer further emphasizes the importance of both the TM12/TM12 interface and the TM9,TM12/TM9,TM12 interface in a biological context for LeuT itself, but also for hDAT and hSERT and potentially also for hNET.

## Discussion

As with all scientific studies, this one is also not without its limitations. The main concerns are linked to the reliance on homology modelling as well as the inherent limitations of computational techniques such as CG MD and free energy calculations^40^. The protein model used in this study has been thoroughly validated in a previous publication and whilst the stickiness of the MARTINI model could cause artifacts^40^, we fortunately see both binding and unbinding events, increasing the reliability of our results. Free energy calculations using the MARTINI model should be guiding, providing trends across systems to emphasize that the values represented here are not absolute but relative energies of dimer dissociation. Sampling issues may arise in all MD studies and to avoid this we made use of both the MARTINI and atomistic models. Fully equilibrating complex systems with membranes can be challenging due to the slow lipid dynamics under our time scale and membrane heterogeneity. In this work we do not see fully equilibrated protein–protein interfaces possibly due to the presence of lipids, and thus it is possible that we miss relevant interfaces as observed for the hSERT self-assembly simulations (Fig. 6e). Nonetheless, it is beyond current computing capabilities to achieve extreme convergence within such complex systems and we therefore rely on this method of ranking interfaces based on the available timescales, which has been shown to reproduce experimentally relevant interfaces^5^. One can also notice the obvious lack of asymptote at long distances in the binding free energies. It has been taken into account but is not as a major limitation. 

In summary, we have presented several probable hDAT and hDAT-palm interfaces primarily involving TM9 and/or TM11, and/or TM12, in good agreement with the LeuT crystal structure dimer and FRET data^24,25^. Specifically, the interfaces conserved among the two systems are TM9/TM9, TM4,TM9/TM11, TM4,TM9/TM2, TM9,TM12/TM9,TM12, TM12/TM4, and to a lesser extent TM12/TM12. The hDAT dimers with the interfaces TM12/TM12, TM9/TM9, TM4,TM9/TM11 and TM9,TM12/TM9,TM12, were further evaluated using US-REMD for separating the two protomers and generating PMF free energy profiles^14^. The four interfaces separation energy resulted in similar PMF values (Fig. 3a) to what has previously been published for other dimer proteins, indicating that these interfaces could potentially be biologically relevant^14,36^. Furthermore, we have shown that the stability of the interfaces involving TM12, TM12/TM12 and TM9,TM12/TM9,TM12, change when hDAT is palmitoylated, suggesting that palm may change the relative abundance of dimers involving TM12 in a biological setup. We anticipate that changes of the membrane composition, e.g. inserting cholesterol, might have similar effects on protein association. The particularity of lipids properties, especially cholesterol, will directly or indirectly affect protein–protein interactions by altering membrane mechanical properties.

According to cysteine cross-linking experiments, TM6/TM6 and TM4/TM4 supposedly form a dimer interface in DAT^20,22^. The authors show by reintroducing the residues Cys243 (in TM4) or Cys306 (in TM6) in cysteine depleted DAT proteins that cross-links are formed across DAT molecules, suggesting that these endogenous cysteine residues located in TM4 and TM6, respectively, are situated at a symmetrical dimer interface^22^. However, in our simulations TM4/TM4 and TM6/TM6 interhelical contacts only rarely form (Fig. 1c). Specifically, the cross-linking residues Cys243 and Cys306 are only found to be symmetrically in contact with the same cysteine in another protomer 0.0% and 0.4% of the time in the hDAT simulations, respectively, and 0.0% and 1.5% in the hDAT-palm simulations, respectively. Nonetheless, this does not necessarily disregard the relevance of our findings. Like all methods, cysteine cross-linking is not without its caveats. A cross-link between proteins will form irrespective of whether the dimer interface is strong or not, as long as the reactive cysteine residues come in to contact with each other during random diffusion, the covalent bond will form and keep the two monomers together.

Similarly, a hDAT dimer was recently constructed using the ClusPro protein–protein docking webserver that contained a C2 symmetrical TM2,TM6,TM11/TM2,TM6,TM11 interface where the two Cys306 residues are in close contact with each other^23^. However, it should be noted that the ClusPro program has primarily been optimized for proteins in solution and the authors state that when docking homology models even moderate errors in key residue side chains or loops may substantially reduce the accuracy of the docking results^41^. It is therefore important to thoroughly review the results from several studies when determining biologically relevant protein dimer interfaces.

Based on our results we propose that a hDAT dimer consisting of TM9/TM12 contacts could be biologically relevant. From both self-assembly simulations and subsequent PMF calculations it is observed that the symmetrical TM9,TM12/TM9,TM12 dimer is energetically favorable. What makes this interface further interesting is that the contacts resemble that of the LeuT crystal structure dimer and can further be reproduced by LeuT self-assembly simulations^25^. Furthermore, it involves interfacial lipids getting encapsulated between the two protomers and it is regulated by a TM12-palm group as shown by the estimation made from PMFs calculation. Lipids involvement in oligomer formation is becoming increasingly studied^14,26,27^. In fact, it has recently been shown by Gupta et al. that the LeuT dimer contains interfacial lipids consisting of one cardiolipin and six phospholipids^26^. When LeuT is in the presence of a dilipidating detergent only the monomeric state is formed, suggesting that the lipids are necessary for dimer formation. It is possible that this could be extended to DAT.

In agreement with both hSERT experimental^24^ and computational^14^ data, we observe that hDAT forms an energetically stable interface consisting of TM12/TM12 interactions. From experimental FRET studies of SERT double TM fragments, it has been shown that the TM11-12 fragment forms a symmetrical interface^24^. This has also been reproduced by computational hSERT self-assembly simulations in which the TM12/TM12 interface was the most energetically favorable^14^. In our study, we observe hDAT TM12/TM12 dimers, although when TM12 becomes palmitoylated the frequency of TM12/TM12 interprotein contacts drops drastically and the free energy of association is decreased. Illustrating how the S-palmitoyl group can have a regulatory effect on hDAT dimer formation.

Jayaramen et al. have published results on hDAT oligomerization using the MARTINI 2.2 FF, but with a different approach to ours, coined the DAFT protocol^15^. Rather than studying systems containing multiple proteins as presented here, they used the DAFT protocol in which multiple repeat simulations are performed of two hDAT proteins randomly orientated in a POPC membrane^15^. We ran 10 repeats of the hDAT and hDAT-palm system each lasting 30 µs, whereas Jayaramen et al. simulated 512 repeats each lasting 2 µs. The DAFT and 16 proteins in a membrane approach have previously converged on similar results for rhodopsin when studying dimers of high occurrence^42^. For hDAT, there are some similarities in the top dimer candidates predicted using the two methods, but overall they differ. The conserved interfaces are TM9/TM9 and EL2/EL2 (detected in our self-assembly, but only presented in Supplementary Fig. S5-6). Jayaramen et al. frequently observe symmetrical dimers containing C-terminal helix contacts, which is somewhat similar to our TM12/TM12 interface with the exception of the two protomers being further separated and thereby not exhibiting direct TM12 contacts. In addition, they observe interfaces involving EL2 loop contacts in three out of eight cases. These interfaces could potentially be biologically relevant, although when using the MARTINI model an elastic network is usually applied, which constraints natural loop flexibility. Thus, different dimer conformations involving loop contacts will inevitably arise depending on the conformation in which the loop has been constrained in. We therefore propose that such contacts should be regarded with care, especially as hDAT presented here and in Jayaramen’s study are homology models. The overall conclusion drawn by the authors was that dimer contacts primarily involve the scaffold region of hDAT (TM 3-5, 8-12) rather than the bundle region (TM1-2,6-7). Although we see different dimer orientations in our simulations, the overall conclusion drawn by Jayaramen et al. also agrees with our results. Finally, we typically first detected full closure of the TM9, TM12/TM9, TM12 interface after ~ 10 µs. Therefore, the same interface cannot be expected to be observed by Jayaramen et al., who only simulated for 2 µs.

The different dimers of high population identified for hDAT and hDAT-palm are similar to those observed for hSERT by Periole et al.^14^. From hSERT self-assembly simulations the top clusters consisted of dimer conformations with TM12/TM12, TM7/TM12, TM4,TM9/TM11,TM2, and finally EL2/EL2 interfaces. Similar interfaces to these have also been observed among the conformations in the top clusters for either hDAT (see Supplementary Fig. S5) or hDAT-palm (see Supplementary Fig. S6) dimers. Interestingly, the separation energy for the hSERT TM4,TM9/TM11,TM2 interface resulted in ~ 20 kJ/mol, whereas the similar hDAT TM4,TM9/TM11 interface resulted in a much higher value of ~ 60 kJ/mol. The TM12/TM12 separation energy for hDAT and hSERT are approximately the same ~ 60 kJ/mol. The TM9/TM9 interface observed frequently in both hDAT and hDAT-palm self-assembly simulations was not among the top dimer clusters of the hSERT self-assembly simulations. A possible explanation for the TM9/TM9 interface being frequently observed for hDAT, but not for hSERT, could be that TM9 in hDAT contains a leucine zipper motif, which is not present in hSERT.

## Conclusion

In conclusion, several energetically favorable hDAT dimer interfaces are accessible and form in a model membrane on a µs time scale. The majority of these dimers contain TM9 and/or TM11 and/or TM12 at the interface, all of which have been experimentally shown to be located at the MAT dimer interfaces^24,25^. Furthermore, we have shown that the energetic changes for interfaces involving TM12 upon hDAT being S-palmitoylated on Cys581, due to palm interfering with the formation of non-palm interprotein contacts. Palm may therefore change the propensity for different dimers that are generated in a biological setting. Overall, it is detected that hDAT and hSERT form similar interfaces and that both the TM12/TM12 and the TM9,TM12/TM9,TM12 interface share a high similarity with the LeuT crystal structure dimer.

The indication that palmitoylation has a modulatory role on dimer formation in hDAT could have numerous biological implications both in terms of protein function and protein surface expression. The regulation of dimer interfaces through S-palmitoylation may alter the reuptake of dopamine from the synaptic cleft or the increased affinity for rafts as a result of palmitoylation may aid in the clustering of hDATs. The exact impact on function however is difficult to assess, mutation studies and an interdisciplinary approach would be highly beneficial to understand this fundamental process.

## Methods

### Homology Modelling and System setup – 16 proteins in a membrane

The data presented here is produced using a hDAT homology model built using a dDAT template (PDB ID: 4XP1). A detailed description of the model building and validation process can be found in an earlier publication^43^. Substrate and coordinating ions were all removed from the atomistic hDAT protein prior to converting it into a MARTINI-v2.2 coarse grain model using the martinize script^44^^.^ The CG protein was inserted into a pure POPC membrane bilayer using the insane script^45^. The MARTINI force field for lipids^46^ and its extension to proteins^44,47^ was used in combination with the ElNeDyn^48^ approach using a 0.9 nm cut-off and a 500 kJ/mol/nm^2^ force constant for maintaining the secondary and tertiary structure of the protein. A single hDAT molecule embedded in a POPC membrane in a 1:99 ratio was copied on a four-by-four grid resulting in a system with 16 hDAT molecules. The orientation of hDAT was randomized by applying a 1000 kJ/mol position restraint on a central residue (BB bead of Leu322) and running a 3.5 µs simulation. Frames at 3.1, 3.2, 3.3, 3.4 and 3.5 µs were selected as starting points for a 30 µs simulation, in which the position restraint was removed, and new velocities were generated with a random seed. Two repeat simulations of each starting frame were conducted resulting in ten repeats totaling in 300 µs simulation time. The same procedure was applied using hDAT S-palmitoylated at Cys581 and LeuT (PDB ID: 2A65). The incorporated parameters for S-palmitoyl have recently been published^49^. In the LeuT self-assembly simulations, a POPE membrane was used instead of a POPC.

### System setup – PMF calculations

The CG dimers used for calculating PMF profiles of hDAT, hSERT, and hDAT-palm interfaces were extracted from the self-assembly simulations and first equilibrated in a POPC membrane of dimensions ~ 9 × 19 × 11 nm^3^ and solvated with water and 0.15 M NaCl. Prior to running the US-REMD simulations the systems were equilibrated by applying a position restraint on each hDAT molecule such that the proteins remained in the center of the short side of the simulation box (10 kJ/mol on the x-axis of the BB bead of Leu322). The dimer interfaces were probed using the distance between the two protomers as the reaction coordinate and simultaneously restraining the dimers relative orientation using the ϕ1 and ϕ3 angle (see Fig. 2a). The distance, ϕ1 and ϕ3 angles where defined using the BB beads of Leu322, Ile393 and Thr456 in hDAT, corresponding to a/A, b/B and c/C, respectively, in Fig. 2a. All dimers at each umbrella started from a bound conformation and became gradually more unbound with increasing distance of separation, d. The umbrella windows were generally separated by 0.1 nm, although in some cases this resulted in a low number of exchanges (> 10%) and a smaller spacing was therefore introduced between these windows (see Supplementary Table S1-2). Ideally, convergence in this type of setup should be evaluated by starting the proteins from both an unbound and a bound conformation and observing the PMF profiles approaching each other. However, as Periole et al. showed for hSERT^14^, the unbound conformation never seemed to approach the bound, due to lipids getting stuck at the interface. Instead, we evaluated system convergence by looking at the PMFs in µs simulation windows (see Supplementary Fig. S22-23). When these approached each other, we concluded the systems to be converged. Typically, this occurred after 3 µs and therefore only the last 7 µs were used for generating the PMFs. Using the distance distributions in the different umbrella windows, the PMF curves were generated using a bootstrap procedure in combination with an in-house implementation of WHAM that takes into account the use of multiple restraints (see Supplementary Fig. S24-25 for the distance histograms). For future unbiasing work the authors recommend using pyemma^50^.

### Molecular dynamics simulations

All MD simulations were performed using the GROMACS simulation package version 5.1.2 compiled with plumed version 2.1^51,52^. The systems containing 16 hDAT molecules were simulated using a 20 fs time step during the production run. The electrostatic and van der Waals non-bonding interactions were treated using a 1.1 nm cut-off and were shifted to 0 using the potential-shift-Verlet scheme as implemented in GROMACS. A dielectric screening constant of 15 was applied. The pressure was regulated at 1 bar semi-isotropically using the Berendsen barostat with a 2.0 ps coupling time constant and the compressibility set to 3 × 10^–4^ bar^-1^. For regulating the temperature, the solvent, protein and membrane were coupled independently to an external heat bath set to 310 K using the velocity rescaling scheme and a 1 ps coupling constant.

The PMF simulations were performed using a slightly different approach. The time step was 20 and 10 fs during equilibration and production runs, respectively. The long range electrostatics were treated using the reaction-field method^53^. Furthermore, the Berendsen barostat was only applied during a 1 µs equilibration step and instead the Parrinello-Rahmen pressure coupling was applied during the 10 µs production run^54^. The frames were saved every 100 ps during production runs. Because the dimers started from a bound state, the first 1–3 μs simulation time was necessary for the individual umbrella windows to equilibrate at the different interface distances. The umbrella windows were exchanged every 20 ps.

It is important to keep in mind that the energies (free energies) obtained within the PMF analysis presented here should be evaluated relative to each other and that such free energy calculations may dependent extremely on the method and the environment, here the membrane bilayer. The force field may also affect the results. Thus all our interpretations are relative and should be interpreted with care.

### Clustering

For clustering the different dimers observed in the self-assembly simulation, all possible protein pairs were first extracted and combined into a single trajectory. From this trajectory all frames in which the distance between the center-of-mass of the BB beads of each protomer was above 5.3 nm were removed. All remaining frames were symmetrized whereby coordinates in protein A were swapped with the coordinates in protein B, which allows for symmetrically related dimer pairs to also be considered. Finally, the trajectory containing the non-swapped and swapped coordinates were combined and clustered using the GROMOS clustering algorithm and a 0.4 nm cutoff.

### Contact analysis

For evaluating all contact data the gmx mindist tool was applied. A total of 12 × 16 index groups were generated containing the indices for the residues that constitute the 12 different helices in hDAT for the 16 different proteins, respectively. In all cases a contact was defined when the distance between any two index groups between protomers was below 7 Å.

## Supplementary Information

Supplementary Information

## Data Availability

The datasets generated and/or analyzed during the current study are available from the corresponding author on reasonable request.

## Abbreviations

AA: All atom
CG: Coarse grain
DAT: Dopamine transporter
EL2: Extracellular loop 2
hDAT, hNET, hSERT: Human DAT, NET or SERT is indicated with a ‘h’
LASA: Lipid accessible surface area
LeuT: Leucine transporter
MAT: Monoamine transporter
NSS: Neurotransmitter Sodium Symporter
NET: Norepinephrine transporter
palm: Palmitoylation
PMF: Potential of mean force
SERT: Serotonin transporter
TM: Transmembrane
US-REMD: Umbrella sampling replica exchange molecular dynamics
POPC: 1-Palmitoyl-2-oleoyl-*sn*-glycero-3-phosphocholine
