# Supplementary material for: Effect of palmitoylation on the dimer formation of the human dopamine transporter
Source: Sci Rep. 2021 Feb 18;11:4164. doi: 10.1038/s41598-021-83374-y (PMC7893171; doi:10.1038/s41598-021-83374-y)
Supplement: Supplementary file 1 — Supplementary Information [file 41598_2021_83374_MOESM1_ESM.pdf]

Supplementary Material

Effect of Palmitoylation on The Dimer Formation of The Human Dopamine Transporter

Talia Zeppelin[a], Kasper Busk Pedersen[b], Nils A. Berglund[a], Xavier Periole[a,c*], Birgit Schiøtt[a,b]*

[a] Department of Chemistry, Aarhus University, Aarhus C, Denmark

[b] Interdisciplinary Nanoscience Center, Aarhus University, Aarhus C, Denmark

[c] Current address: Faculty of Science, University of Auckland, New Zealand

* Corresponding author, Birgit Schiøtt, birgit@chem.au.dk, Xavier Periole, x.periole.science@gmail.com

Preparing the all-atom TM9,TM12/TM9,TM12 system

The system studied at atomistic resolution was built from backmapping a TM9,TM12/TM9,TM12 dimer using the backwards script and substituting the proteins with an already equilibrated hDAT all-atom protein containing coordinating ions and bound dopamine. The system was neutralized by adding 0.15 NaCl to the aqueous phase. Due to a flexible extracellular loop (EL2) in hDAT, which is otherwise fixed in the CG system with the elastic network, additional solvent was added to the extracellular side of the box in the AA system. The dopamine parameters applied are given in a previous publication[1]. The final box dimensions were ~13x13x9 nm^3.

Simulating the all-atom TM9,TM12/TM9,TM12 system

When simulating the all-atom hDAT dimer system the GROMACS version 5.1.4[2,3] MD engine was used together with the CHARMM36 force field[4,5]. Protein and lipid H-bonds were constrained using the LINCS algorithm[6,7] and water molecules were constrained using SETTLE[8], which enabled the use of a 2 fs time step. The system was first minimized using the steepest decent algorithm followed by six equilibration steps in which restraints on the protein and lipids were gradually decreased from 1000 kJ/mol*nm^2[9]. For analysis purposes the coordinates were saved every 100 ps during the MD simulation. The temperature was kept at 310 K using a Nosé-Hoover scheme[10] and the pressure

was maintained at 1 atm using a Parrinello-Rahman approach[11] with semi-isotropic scaling. A Berendsen thermo- and barostat was used for equilibration[12]. A cutoff of 12 Å was applied for the van der Waals interactions using a switch function starting at 10 Å[13]. The cutoff for the short-range electrostatic interactions was 12 Å and for computing the long-range interactions the Particle-Mesh Ewald algorithm was applied[14]. The final production run resulted in a 530 ns trajectory.

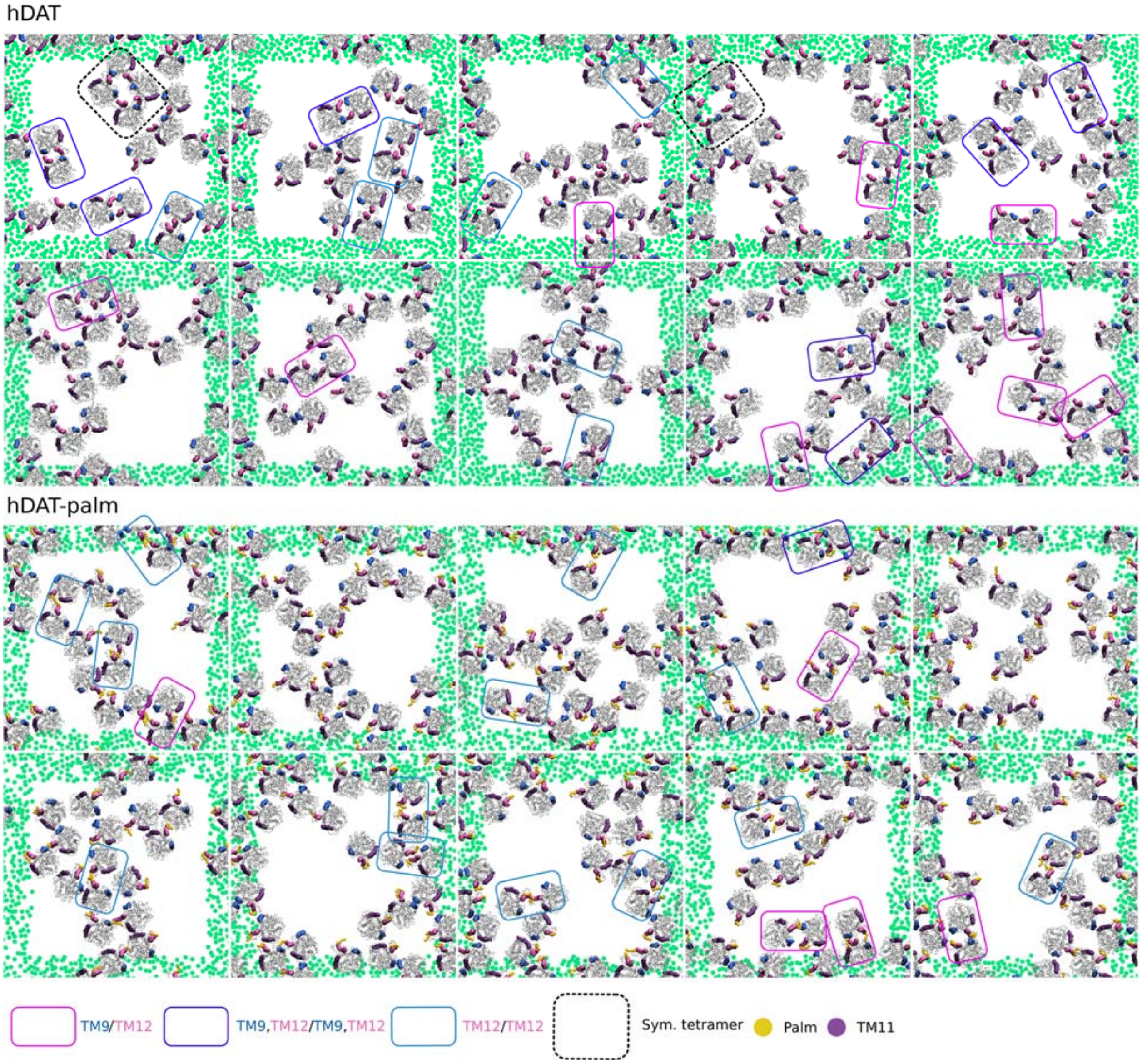

Figure S1: hDAT (top) and hDAT-palm (bottom) self-assembly simulations. Represented are the 10 last frames of the hDAT and hDAT-palm repeat simulations. Each system contains either 16 hDAT or hDAT-palm proteins equally spaced but randomly orientated in a pure POPC membrane and has been simulated for 30 µs. The helices TM9, TM11, and TM12 are highlighted in blue, purple, and mauve, respectively. The palmitoyl group (Palm) is highlighted in orange. Three different conserved dimer interfaces are emphasized by blue, pink and purple boxes. The pink boxes contain a TM9/TM12 interface and illustrated in the purple boxes is a symmetrical interface involving both TM9 and TM12 helices, dubbed TM9,TM12/TM9,TM12. In the blue boxes a TM12/TM12 interface is observed and in the black dotted box a symmetrical tetramer is represented. The green dots correspond to the GL1 bead in POPC and are shown for the nearest periodic images to the system. For the single simulation box the dots have been omitted.

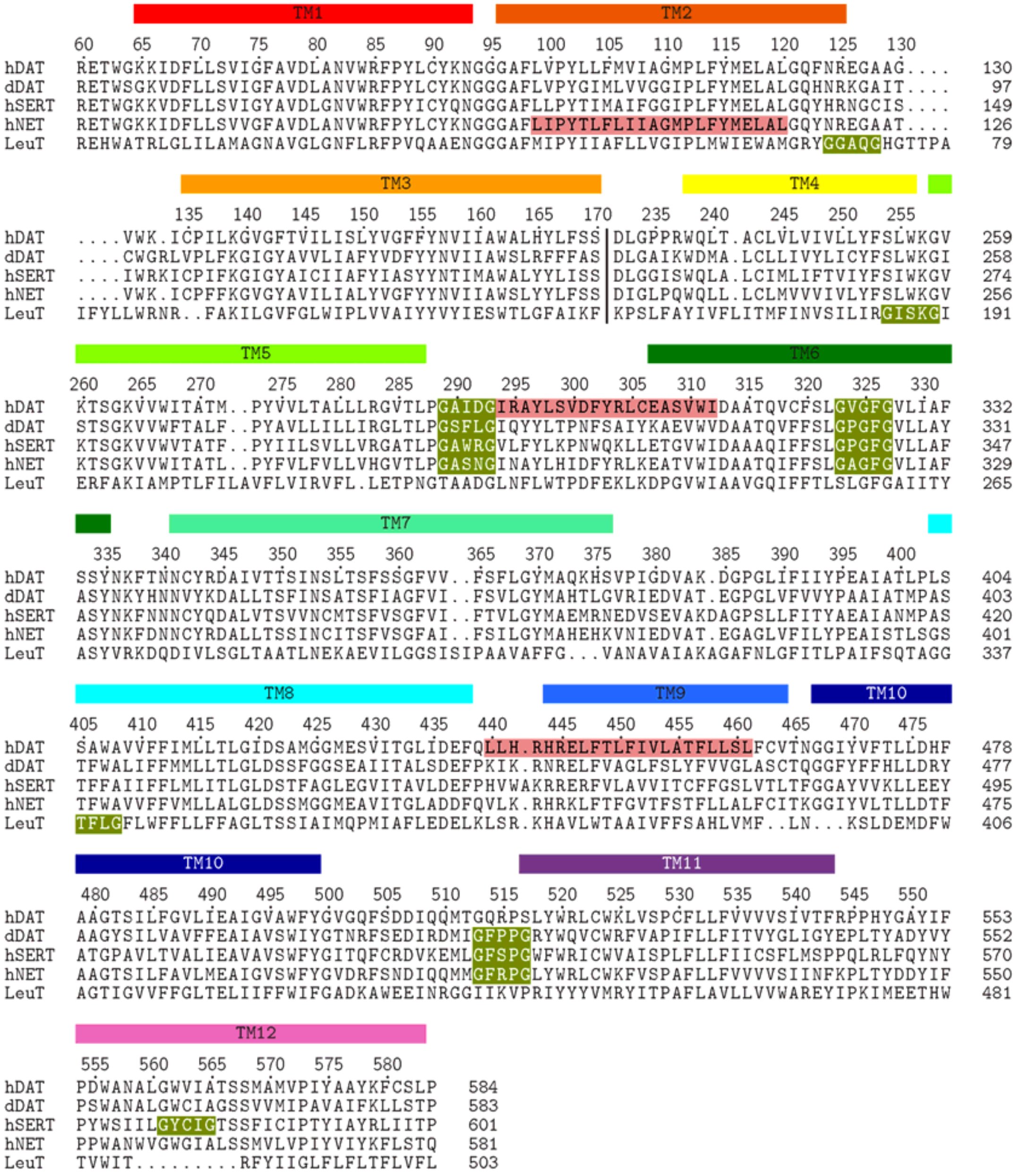

Figure S2: Multiple sequence alignment using Clustal Omega of hDAT (uinprot ID: Q01959), dDAT (uniprot ID: Q7K4Y6), hSERT (uniprot ID: P31645), hNET (uniprot ID: P23975) and LeuT (uniprot ID: O67854). Highlighted in the alignment are the oligomerization promoting motifs: GXXXG (green) and $LX_6LX_6LX_6LX_6L$ (pink). Due to the high similarity between Ile and Leu, leucine zipper motifs containing Ile instead of Leu have also been included. The approximate location of the 12 different transmembrane (TM) helices are indicated above the alignment using the same coloring scheme as applied throughout the manuscript. The alignment has been made using the texshade package in LaTeX[15].

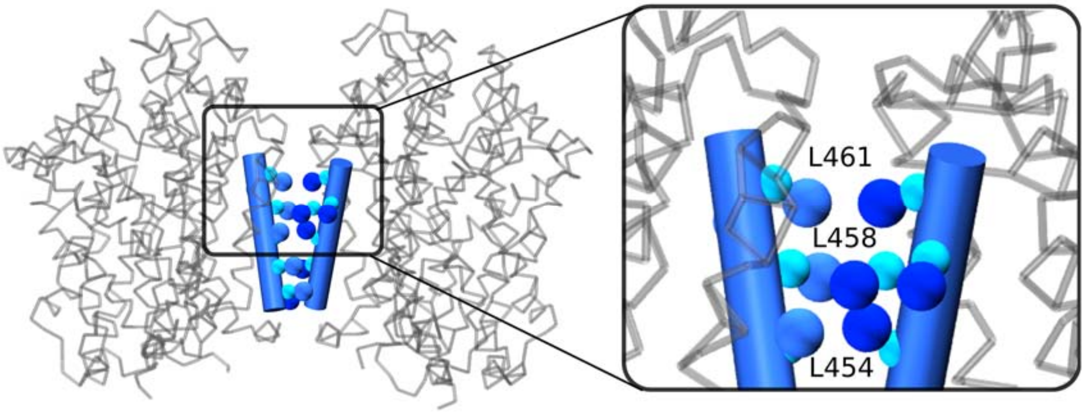

Figure S3: Leucine zipper motif on hDAT. Shown is a representative hDAT dimer in which TM9 on one protomer is interacting with TM9 on another protomer. The highlighted section depicts the three leucine residue pairs which can readily form contacts at the interface. The sidechain beads are colored different shades of blue depending on which protomer they belong to.

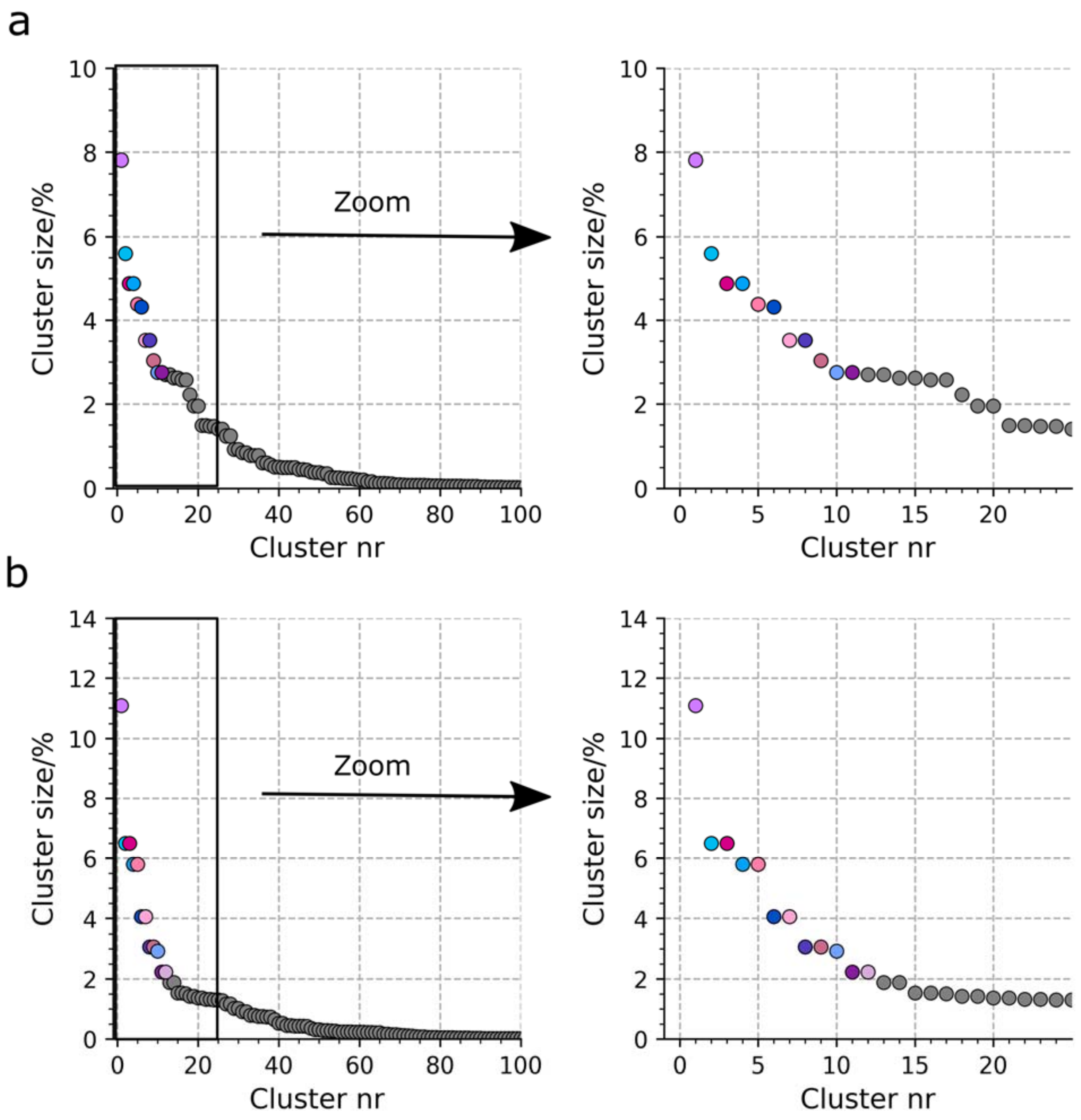

Figure S4: Cluster size in % as a function of cluster number. Plotted in a) and b) are the cluster sizes as a function of cluster number for the different hDAT and hDAT-palm dimers, respectively. The different dot colors correspond to the top clusters, which are also presented in Figure S7.

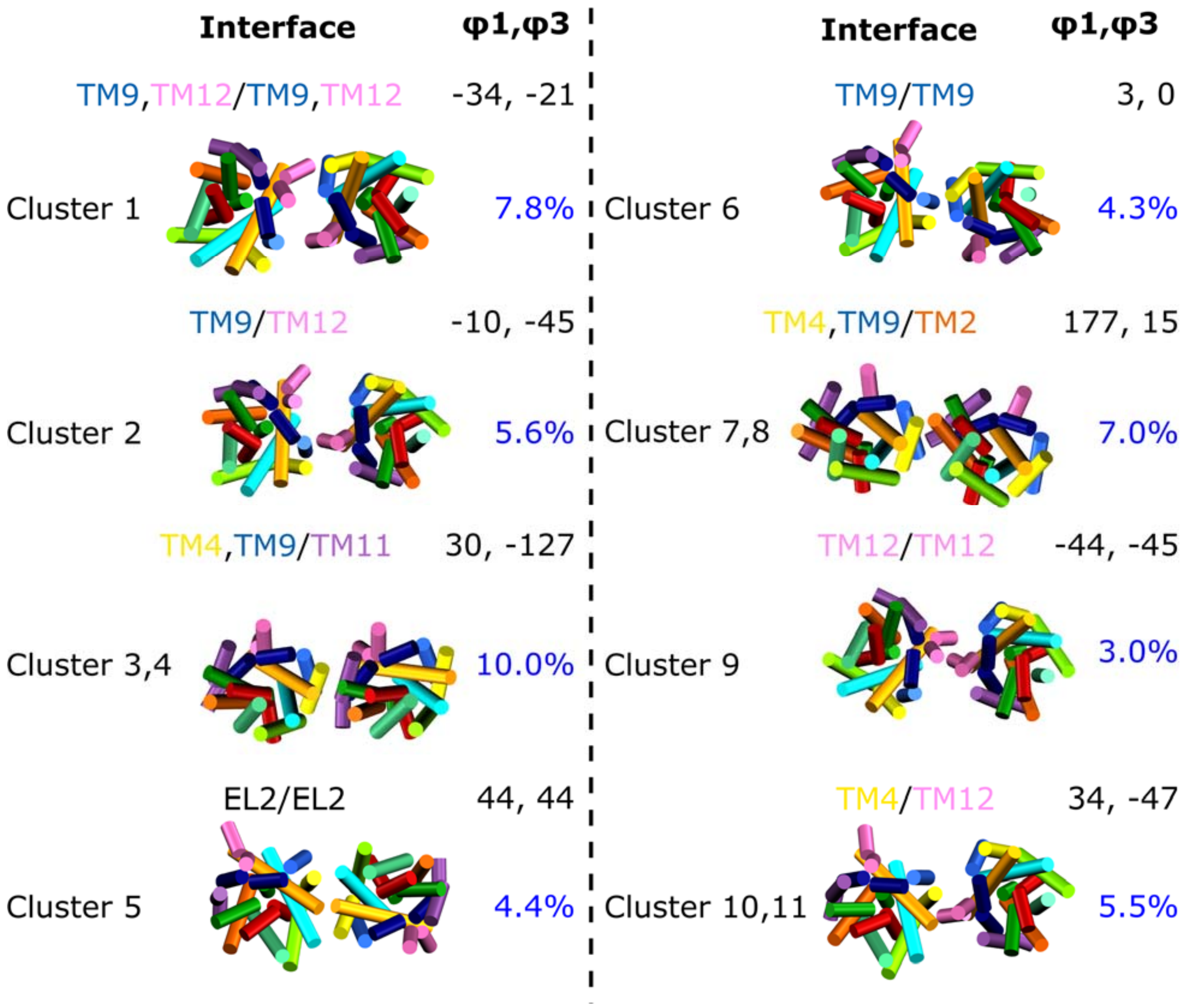

Figure S5: Top hDAT clusters. Represented are the top 11 largest clusters of hDAT dimers extracted from 10 self-assembly simulations of systems containing 16 hDAT molecules embedded in a pure POPC membrane. The representative dimer conformation of the top clusters are depicted using the same TM color scheme as applied in Figure 2a. Listed above the different representative dimers are the helices involved at the interface. Highlighted in blue writing is the size of the different clusters extracted from Figure S4. Clusters with more than one cluster number have been grouped together due to symmetry reasons. The values of the ϕ1 and ϕ3 angles (illustrated in Figure 2a) for the representative cluster dimers are also listed.

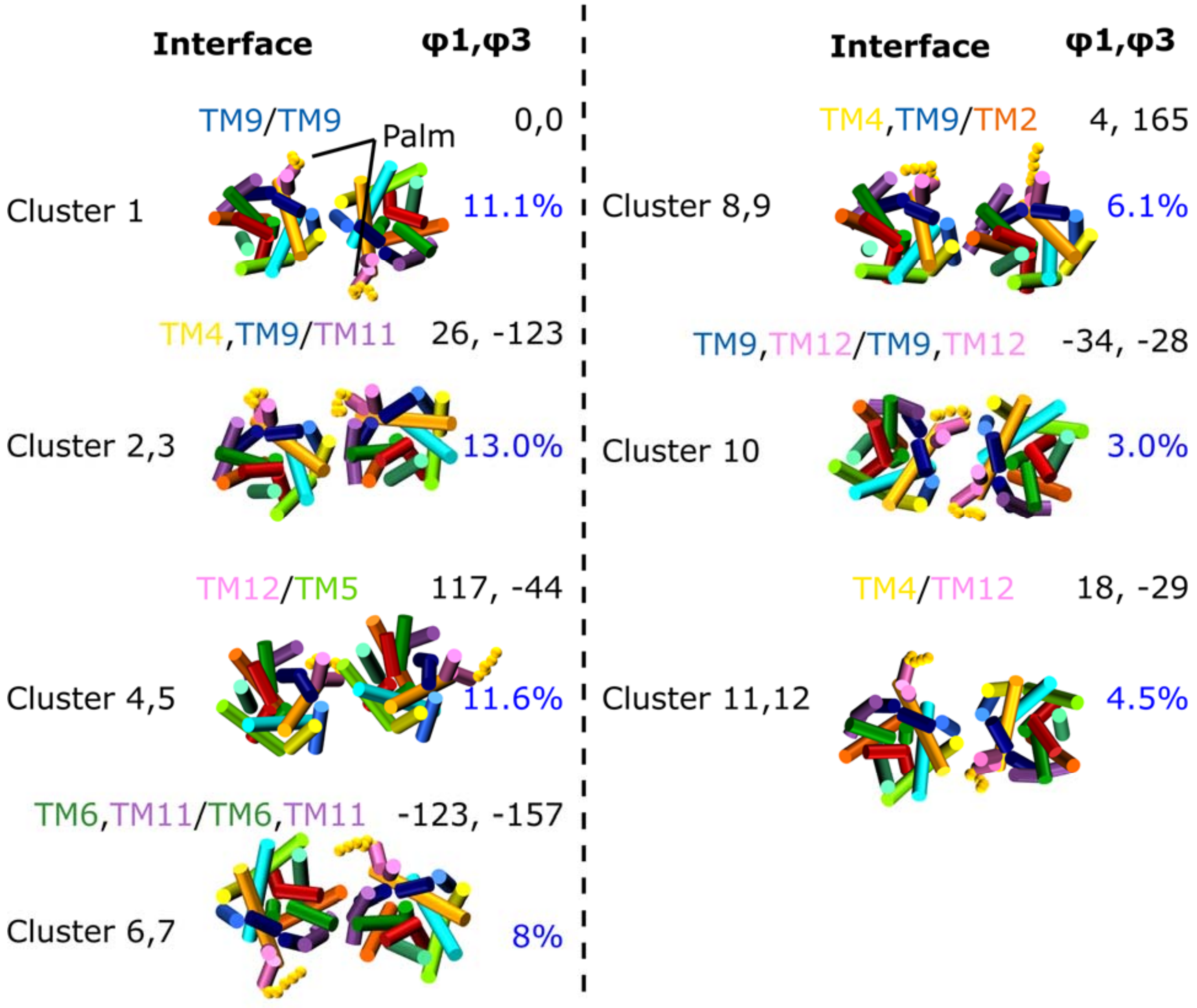

Figure S6: Top hDAT-palm clusters. Represented are the top 12 largest clusters of hDAT-palm dimers extracted from 10 self-assembly simulations of systems containing 16 hDAT-palm molecules embedded in a pure POPC membrane. The representative dimer conformation of the top clusters are depicted using the same TM color scheme as applied in Figure 2a. Listed above the different representative dimers are the helices involved at the interface. Highlighted in blue writing is the size of the different clusters extracted from Figure S4. Clusters with more than one cluster number have been grouped together due to symmetry reasons. The values of the ϕ1 and ϕ3 angle (illustrated in Figure 2a) for the representative cluster dimers are also listed.

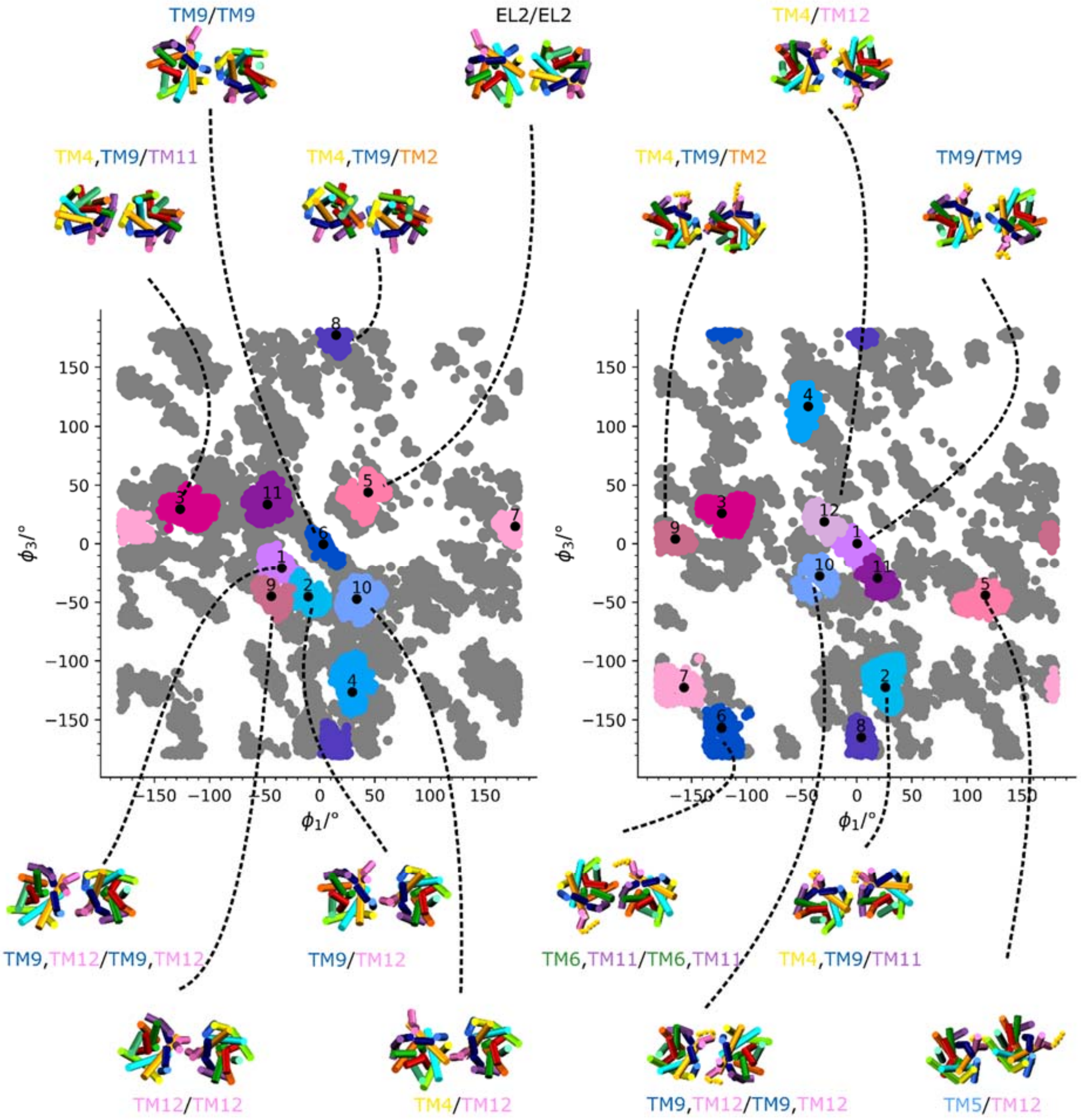

Figure S7: hDAT (left) and hDAT-palm (right) dimers plotted in the ϕ1 and ϕ3 angle space. All the hDAT and hDAT-palm dimers are represented as a dot in the ϕ1 and ϕ3 space using the definition illustrated in Figure 2a). The different colors indicate the dimer conformations that are part of the top clusters (the same colors as applied in Figure S4). The representative dimer conformation of the different clusters, which have the highest number of neighbors, are depicted as black dots. The representative conformations of the different clusters are shown with their contacting helices highlighted.

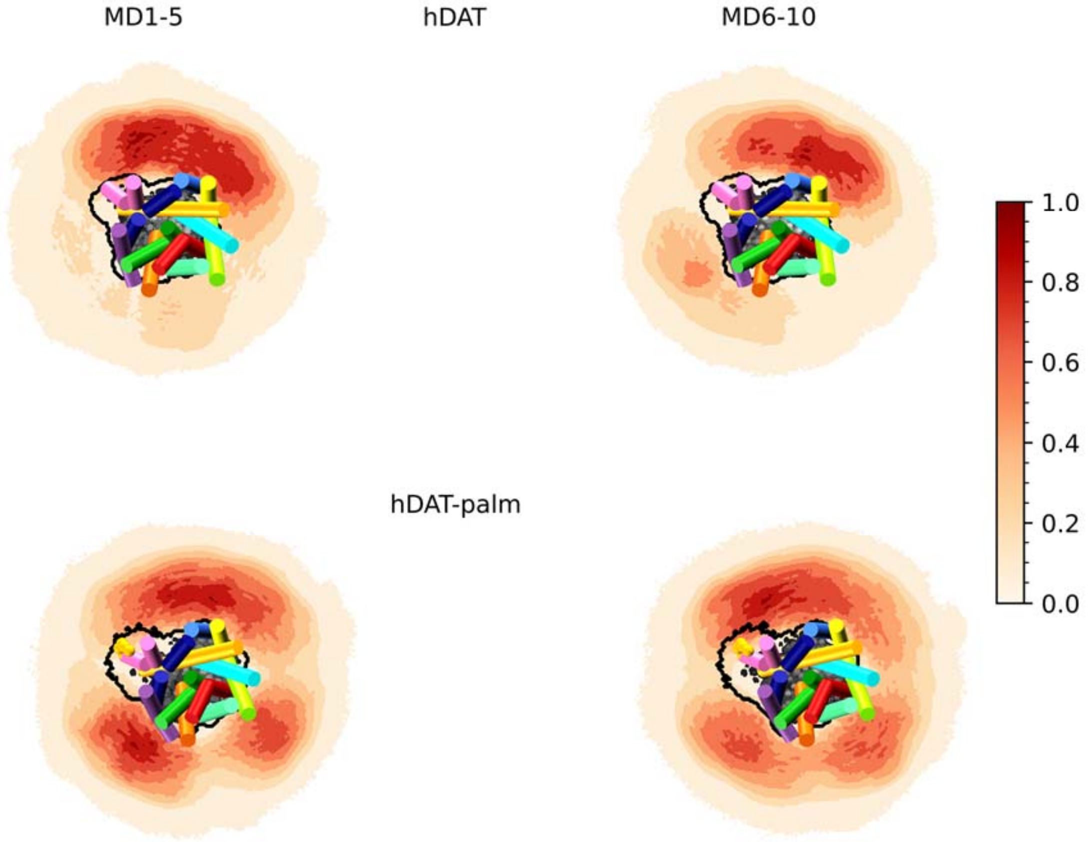

Figure S8: Dimer occupancy maps illustrating that the hDAT and hDAT-palm systems probe similar interfaces, which are quite conserved across repeat simulations. The occupancy maps represent all hDAT and hDAT-palm dimers extracted from the first 5 self-assembly simulations (MD1-MD5/left) and last 5 self-assembly simulations (MD6-MD10/right) in which the dimer trajectories have been fitted on a single protomer. The fitted protein helices are highlighted using the same coloring scheme as given in Figure 2a.

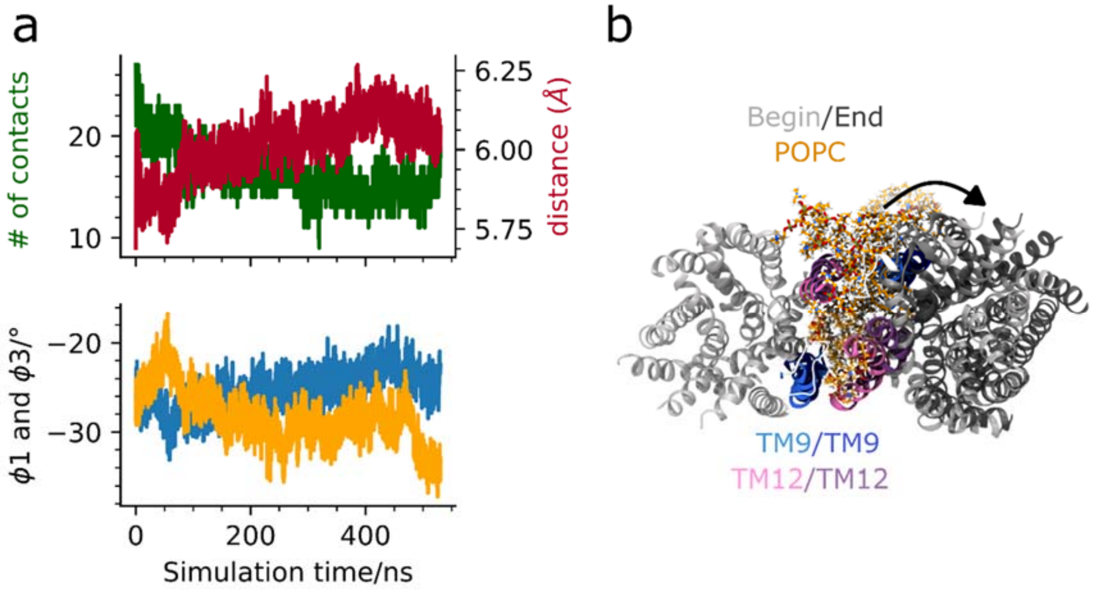

Figure S9: All-atom hDAT data. Represented in the top plot of a) is the number of contacts (green) and the distance of separation (red) with respect to the two protomers as a function of time. A contact was considered when any distance between the two protomers residues was below 5 Å. Depicted in the bottom plot of a) is the $\phi1$ (blue) and $\phi3$ (orange) angle as a function of time. In b) the start and end frame from the all-atom simulation are aligned, illustrating that the single TM9/TM12 interface detaches enabling POPC lipids to enter and exit the interface.

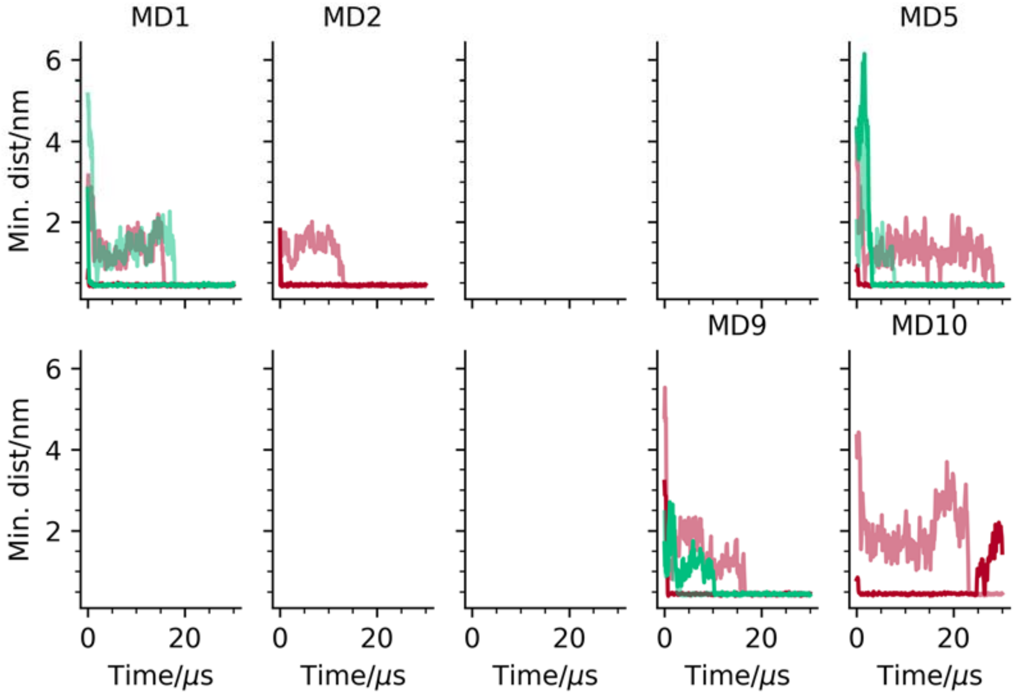

Figure S10: hDAT dimer double TM9,TM12/TM9,TM12 contact as a function of time. For each of the 10 repeat simulations containing 16 hDAT molecules in a pure POPC membrane all TM9,TM12/TM9,TM12 dimers were extracted in which both TM pairs (TM9/TM12 and TM12/TM9) come together for more than 500 ns during the course of the simulation. A contact between helices was defined if the minimum distance was below 7 Å. Each distinct color represents a different TM9,TM12/TM9,TM12 dimer pair, which is further subdivided into two similar color shades representing the minimum distance for each TM12/TM9 helix pair located in the same dimer. Frames were analyzed every 100 ns throughout the 30 µs simulations. Each plot represents a different repeat simulation. Notice, the first TM9/TM12 contact is already formed in most cases during the first 100 ns, while the remaining TM12/TM9 first come together much later. In five repeat simulations no TM9,TM12/TM9,TM12 dimers are formed, which is why five plots are empty.

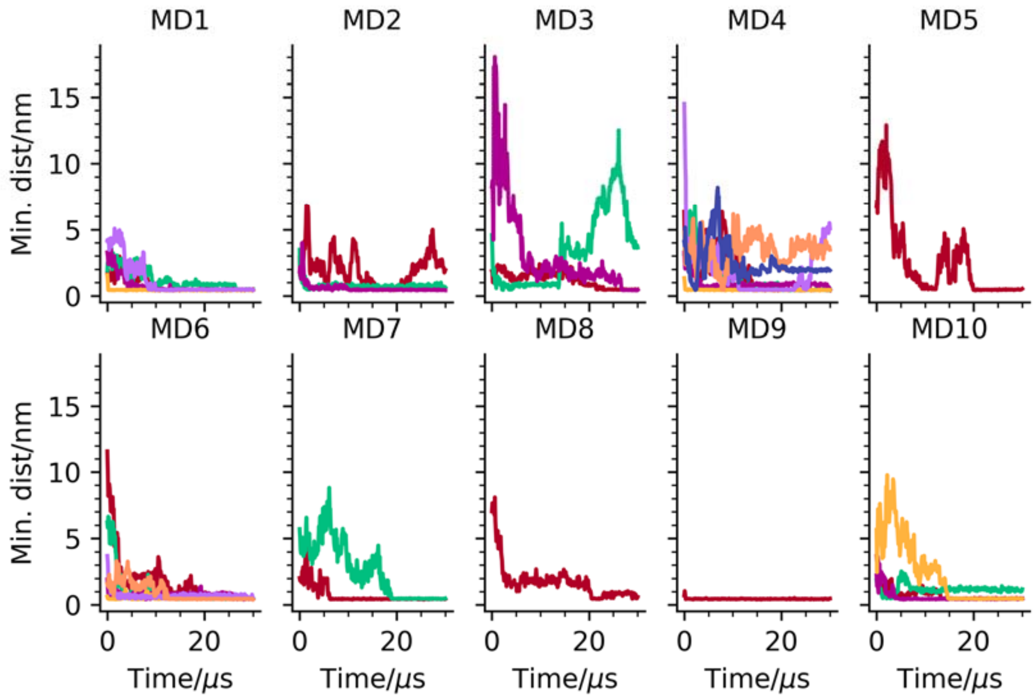

Figure S11: hDAT dimer TM9/TM12 single helix pair contact as a function of time. For each of the 10 repeat simulations containing 16 hDAT molecules in a pure POPC membrane all the dimers were extracted in which only a single TM9/TM12 pair come in to contact for more than 500 ns during the course of the simulation. A contact between helices was defined if the minimum distance was below 7 Å. Each distinct color represents a different TM9/TM12 dimer pair. Frames were analyzed every 100 ns throughout the 30 μs simulations. Each plot represents a different repeat simulation.

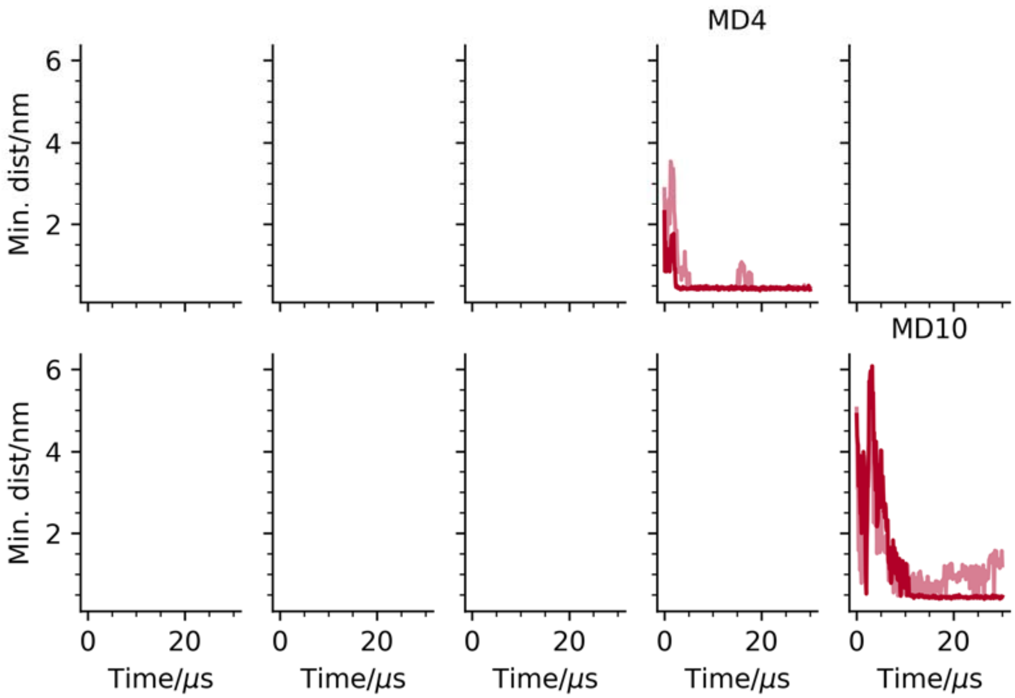

Figure S12: hDAT-palm dimer double TM9,TM12/TM9,TM12 contact as a function of time. For each of the 10 repeat simulations containing 16 hDAT-palm molecules in a pure POPC membrane all TM9,TM12/TM9,TM12 dimers were extracted in which both TM pairs (TM9/TM12 and TM12/TM9) come together for more than 500 ns during the course of the simulation. A contact between helices was defined if the minimum distance iwas below 7 Å. Each distinct color represents a different TM9,TM12/TM9,TM12 dimer pair, which is further subdivided into two similar color shades representing the minimum distance for each TM12/TM9 helix pair located in the same dimer. Frames were analyzed every 100 ns throughout the 30 µs simulations. Each plot represents a different repeat simulation. In eight repeat simulations no TM9,TM12/TM9,TM12 dimers are formed, which is why eight plots are empty.

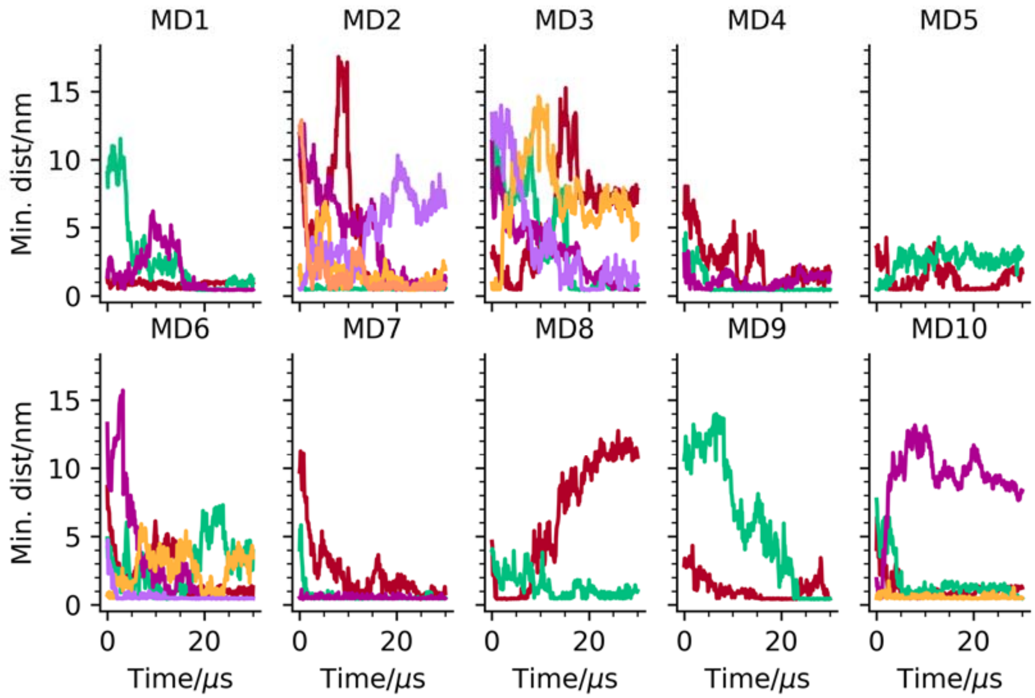

Figure S13: hDAT-palm dimer TM9/TM12 single helix pair contact as a function of time. For each of the 10 repeat simulations containing 16 hDAT-palm molecules in a pure POPC membrane all the dimers were extracted in which only a single TM9/TM12 pair come in to contact for more than 500 ns during the course of the simulation. A contact between helices was defined if the minimum distance was below 7 Å. Each distinct color represents a different TM9/TM12 dimer pair. Frames were analyzed every 100 ns throughout the 30 µs simulations. Each plot represents a different repeat simulation.

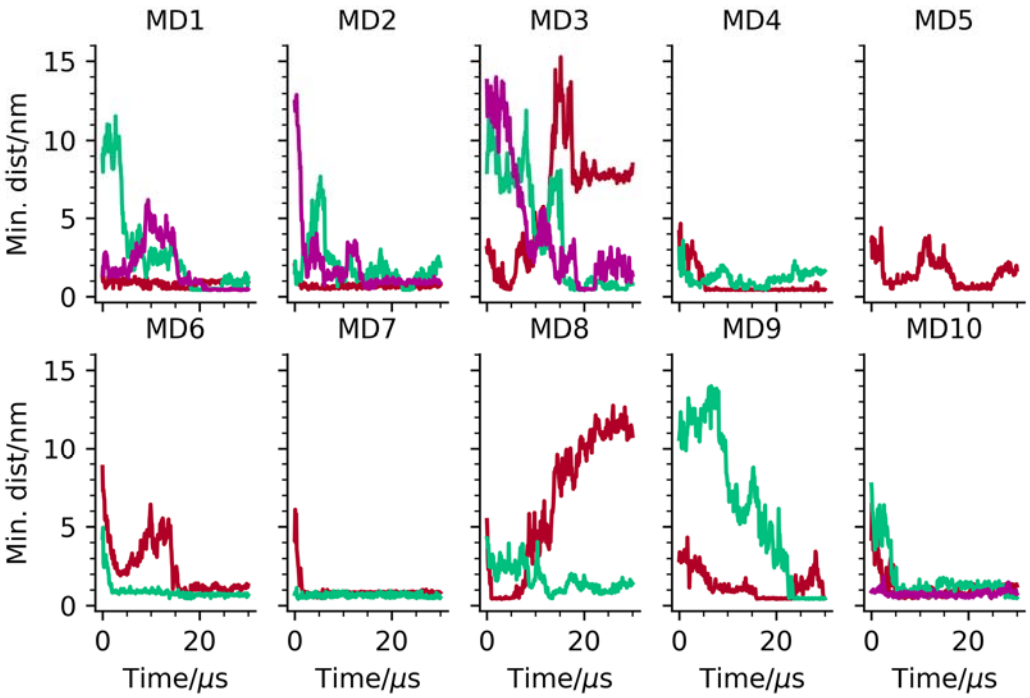

Figure S14: hDAT-palm dimer TM9/TM12 single helix pair contact as a function of time, when excluding palm contacts from the analysis. For each of the 10 repeat simulations containing 16 hDAT-palm molecules in a pure POPC membrane all the dimers were extracted in which only a single TM9/TM12 pair come in to contact for more than 500 ns during the course of the simulation. A contact between helices was defined if the minimum distance was below 7 Å. Each distinct color represents a different TM9/TM12 dimer pair. Frames were analyzed every 100 ns throughout the 30 μs simulations. Each plot represents a different repeat simulation.

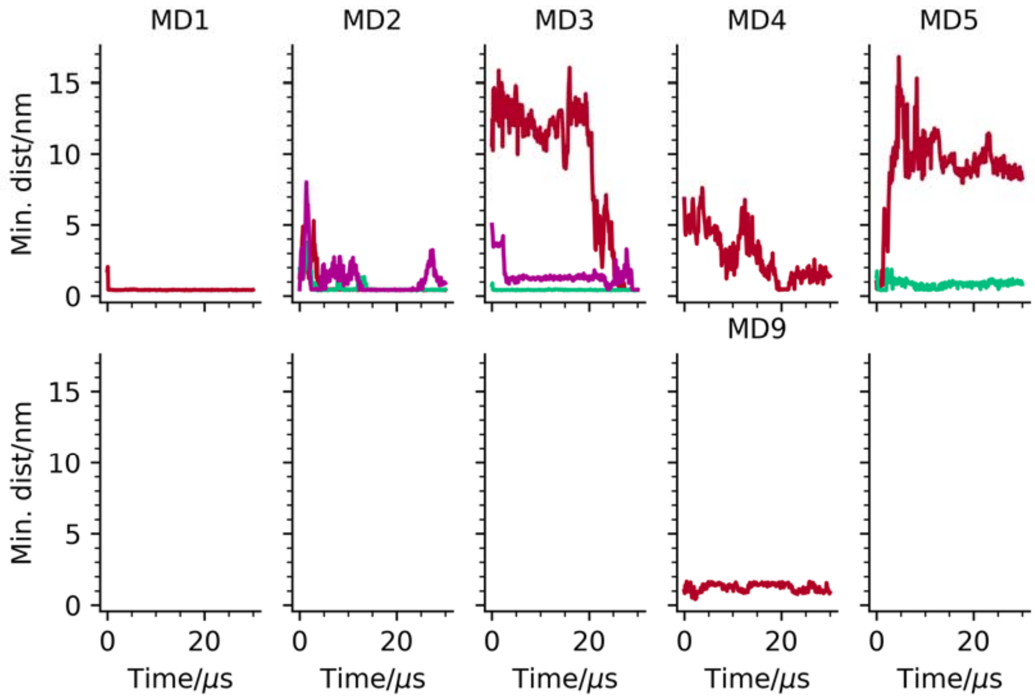

Figure S15: hDAT dimer TM12/TM12 contact as a function of time. For each of the 10 repeat simulations containing 16 hDAT molecules in a pure POPC membrane all the dimers were extracted in which TM12/TM12 come in to contact for more than 500 ns during the course of the simulation. A contact between helices was defined if the minimum distance was below 7 Å. Each distinct color represents a different TM12/TM12 dimer pair. Frames were analyzed every 100 ns throughout the 30 μs simulations. Each plot represents a different repeat simulation. In four repeat simulations no TM12/ TM12 dimers are formed, which is why four plots are empty.

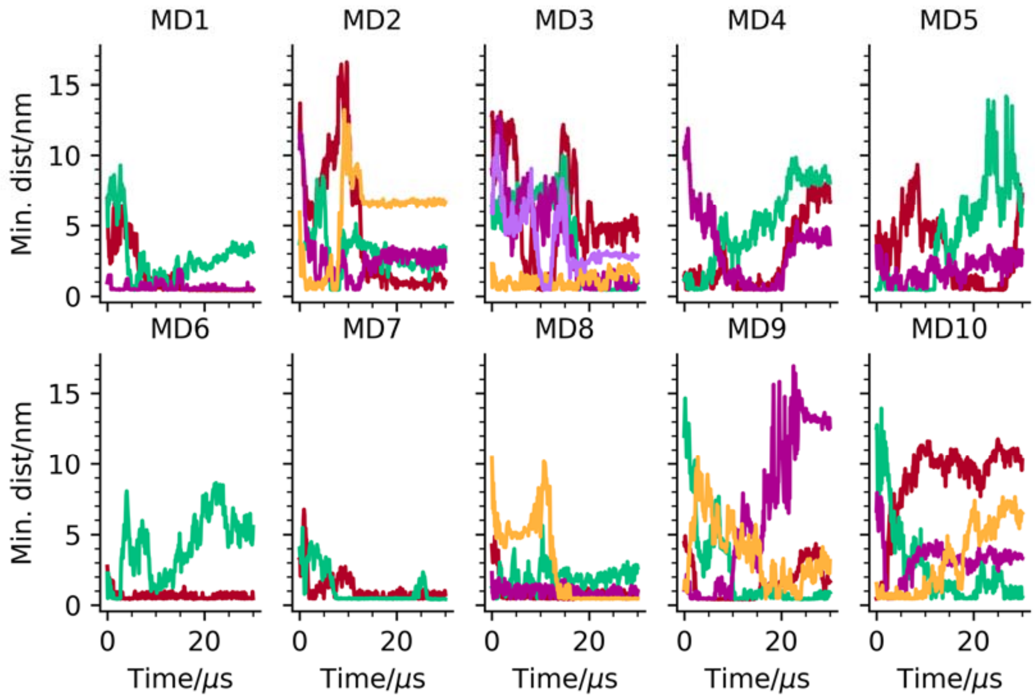

Figure S16: hDAT-palm dimer TM12/TM12 contact as a function of time. For each of the 10 repeat simulations containing 16 hDAT-palm molecules in a pure POPC membrane all the dimers were extracted in which TM12/TM12 come in to contact for more than 500 ns during the course of the simulation. A contact between helices was defined if the minimum distance was below 7 Å. Each distinct color represents a different TM12/TM12 dimer pair. Frames were analyzed every 100 ns throughout the 30 μs simulations. Each plot represents a different repeat simulation.

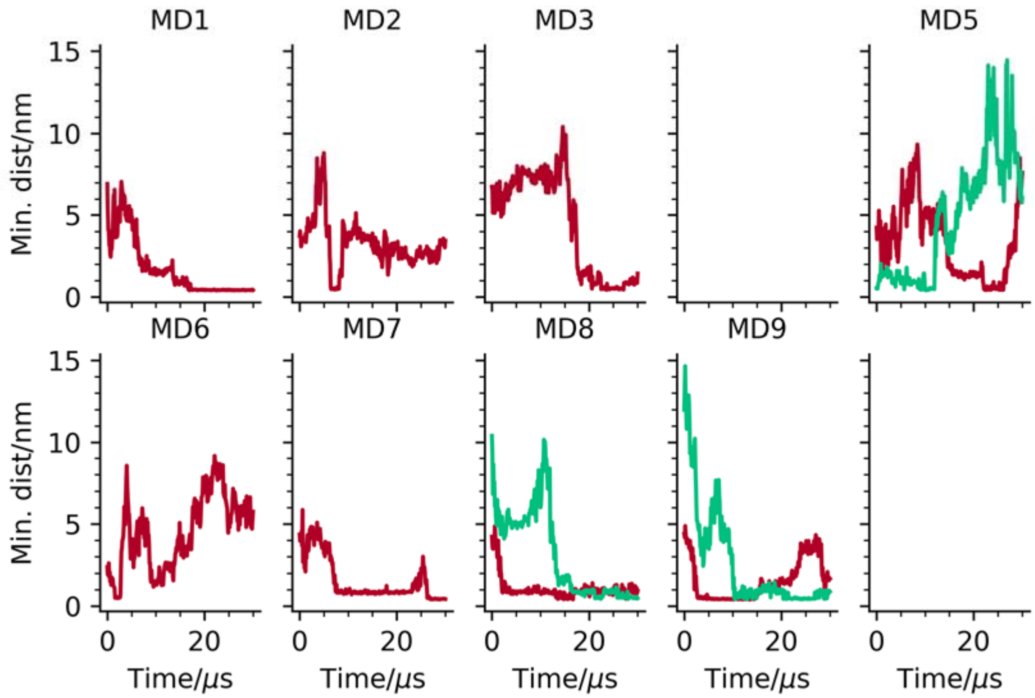

Figure S17: hDAT-palm dimer TM12/TM12 contact as a function of time, when excluding palm contacts from the analysis. For each of the 10 repeat simulations containing 16 hDAT-palm molecules in a pure POPC membrane all the dimers were extracted in which TM12/TM12 come in to contact for more than 500 ns during the course of the simulation. A contact between helices was defined if the minimum distance was below 7 Å. Each distinct color represents a different TM12/TM12 dimer pair. Frames were analyzed every 100 ns throughout the 30 µs simulations. Each plot represents a different repeat simulation. In two repeat simulations no TM12/TM12 dimers are formed, which is why two plots are empty.

LeuT

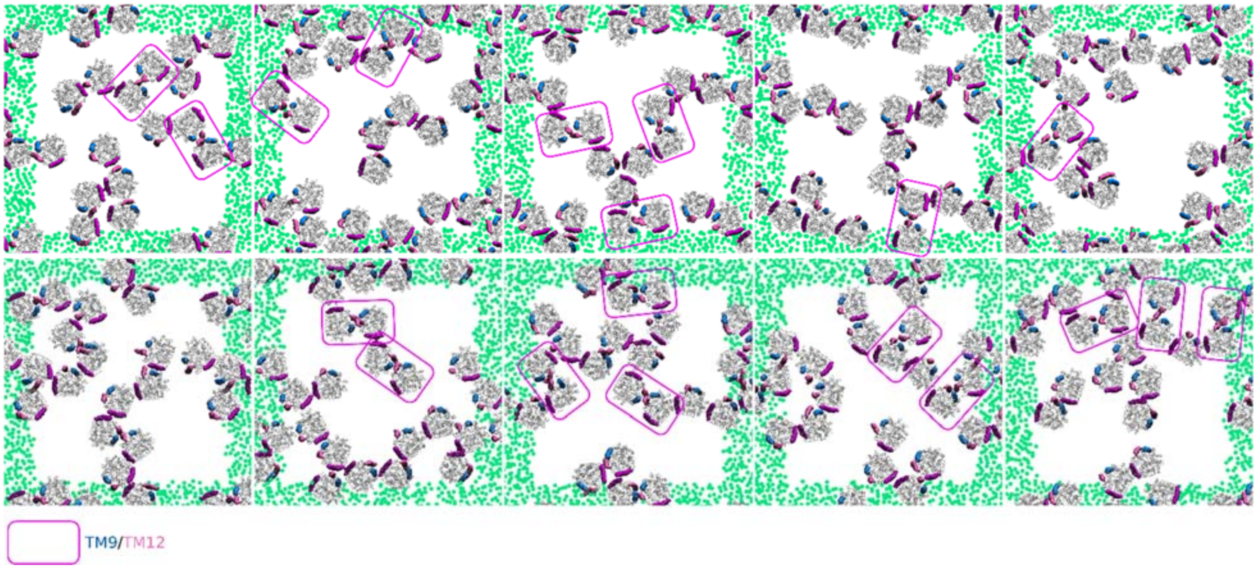

Figure S18: LeuT self-assembly simulations. Represented are the last frames of the 10 LeuT repeat simulations. Each system contains 16 LeuT (PDB ID: 2A65) proteins equally spaced but randomly orientated in a pure POPE membrane and has been simulated for 30 µs. The helices TM9, TM11, and TM12 are highlighted in blue, purple, and mauve, respectively. Emphasized by pink boxes are dimers containing TM9/TM12 interfaces. The green dots correspond to the GL1 bead in POPC and are shown for the nearest periodic images to the system. For the single simulation box the dots have been omitted.

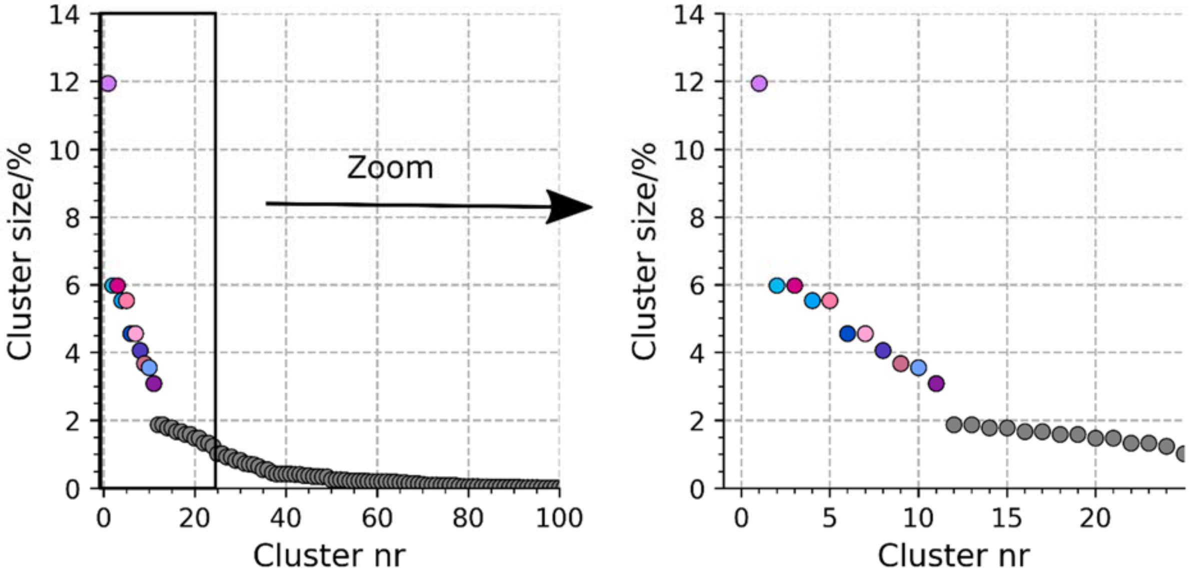

Figure S19: LeuT cluster size in % as a function of cluster number. Plotted to the left is the the size of the different LeuT dimer clusters as a function of cluster number. To the right, a zoom is shown of the same plot. The different dot colors correspond to the top clusters which are also presented in Figure S21.

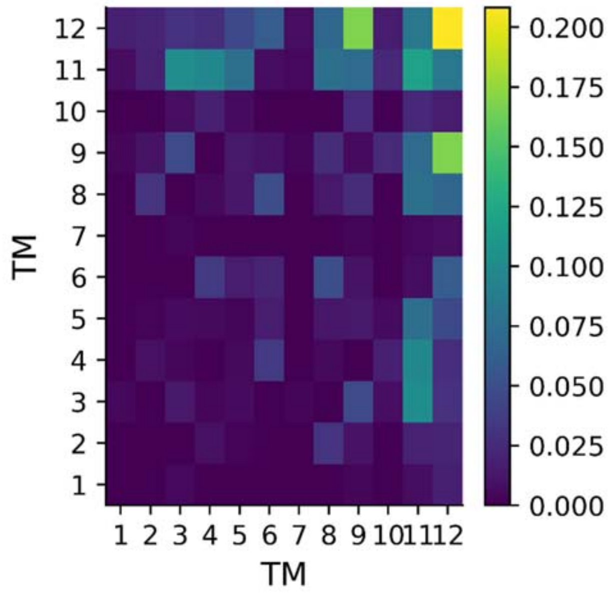

Figure S20: LeuT dimer per helix degree of contact in %. Contact is considered when any residue in the different helices of one LeuT protein is within 7.5 Å of any other residue in the different helices of the other LeuT protein found in a dimer.

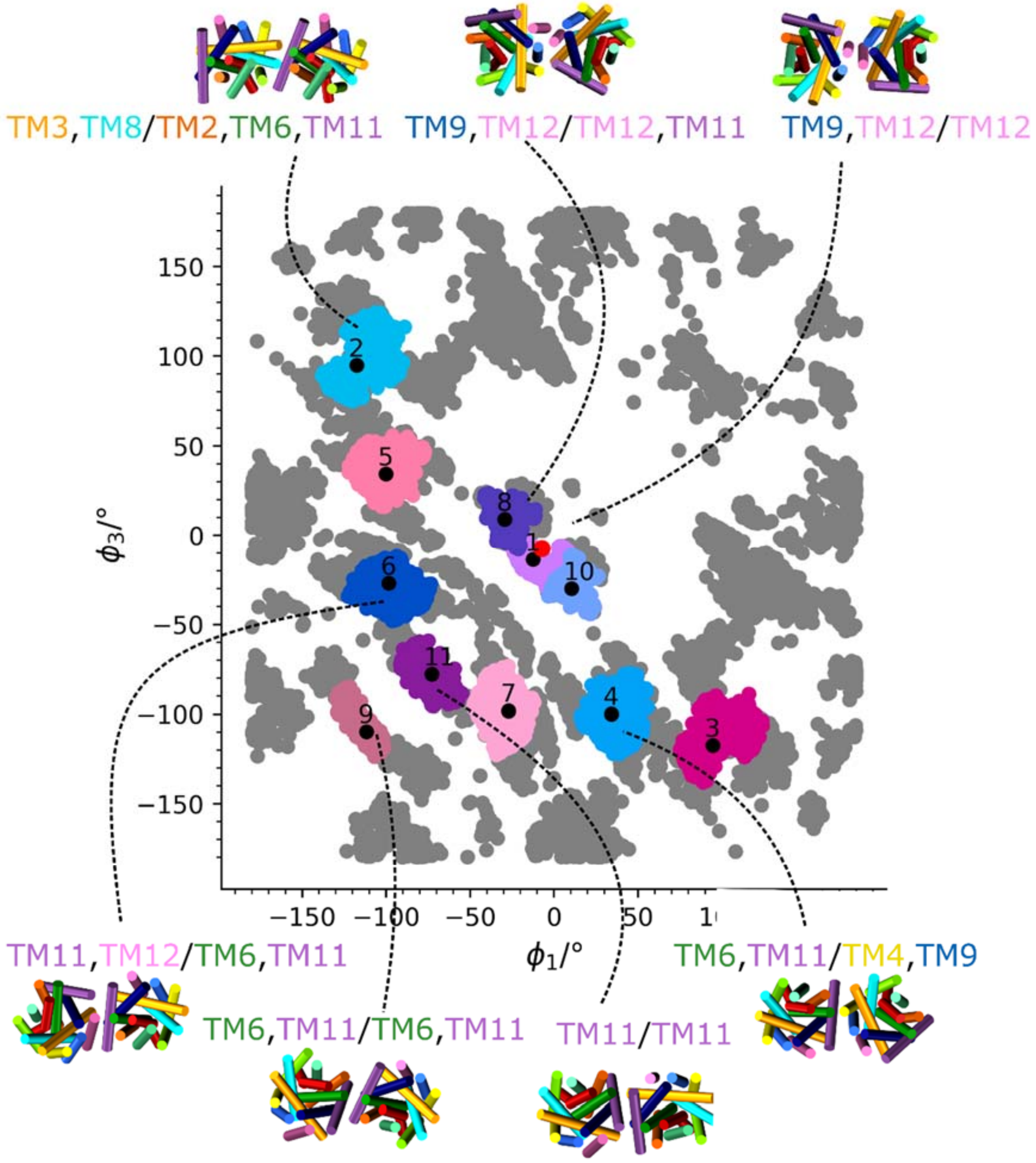

Figure S21: LeuT dimers plotted in the ϕ1 and ϕ3 angle space. All the LeuT dimers are represented as a dot in the ϕ1 and ϕ3 space using the definition illustrated in Figure 2a. The different colors indicate the dimer conformations that are part of the top clusters (the same colors as applied in Figure S19). The representative dimer conformation of the different clusters, which have the highest number of neighbors, are depicted as black dots. The representative conformations of the different clusters are shown with their contacting helices highlighted. The red dot corresponds to the ϕ1 and ϕ3 angle of the LeuT crystal structure dimer (-7,3°/-7,3°).

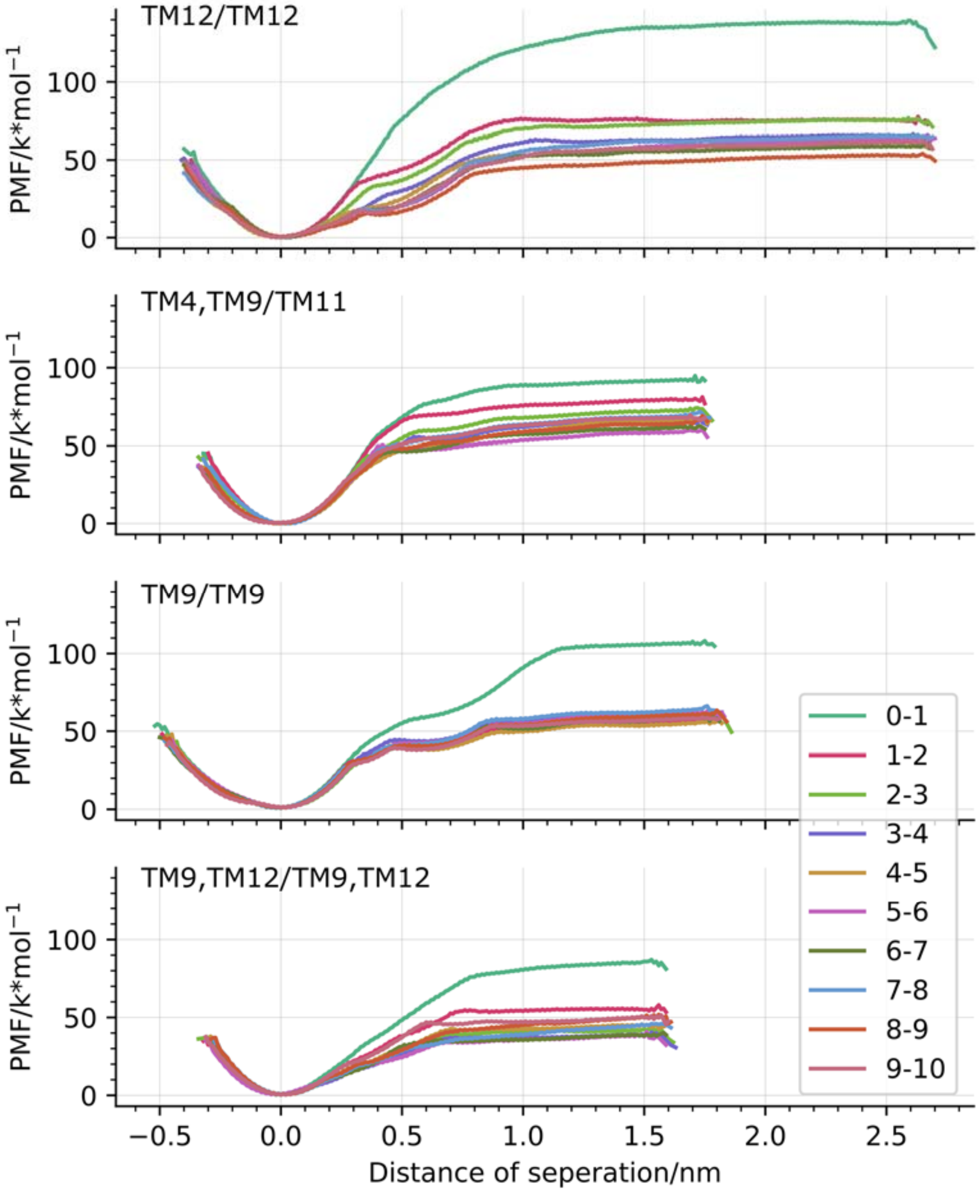

Figure S22: Time-evolution in μs time intervals of PMF profiles for the four different hDAT interfaces studied.

Interface	TM12/TM12	TM9/TM9	TM9,TM12/TM9,TM12	TM4,TM9/TM11
ϕ1 (°)	-42.40	2.06	-27.50	-134.07
ϕ3 (°)	-42.40	2.06	-27.50	26.36
ϕ1, ϕ3 force (kJ/mol)	200.00	300.00	300.00	300.00
distance force (kJ/mol)	1000.00	1000.00	1000.00	1000.00
distance at minimum (Å)	5.30	6.20	5.70	5.00
Simulation used for PMF calculations (µs)	3-10	1-10	2-10	2-10
Umbrella distance (Å)	4.90	5.70	5.40	4.70
	5.00	5.80	5.50	4.80
	5.10	5.90	5.60	4.90
	5.20	6.00	5.70	5.00
	5.30	6.10	5.80	5.10
	5.40	6.20	5.90	5.20
	5.50	6.30	6.00	5.30
	5.60	6.40	6.10	5.40
	5.70	6.50	6.20	5.50
	5.80	6.60	6.30	5.60
	5.90	6.70	6.40	5.70
	6.00	6.80	6.50	5.80
	6.10	6.90	6.60	5.90
	6.20	7.00	6.70	6.00
	6.30	7.10	6.80	6.10
	6.40	7.20	6.90	6.20
	6.50	7.30	7.00	6.30
	6.60	7.40	7.10	6.40
	6.70	7.50		6.50
	6.80	7.60		6.60
	6.90	7.70		
	7.00	7.80		
	7.10			
	7.20			
	7.30			
	7.40			
	7.50			
	7.60			

Table S1: Overview of the settings applied in the US-REMD hDAT simulations. Listed are the ϕ1 and ϕ3 angle values and their restraint force constant for the different interfaces. In addition, the distance restraint applied at the different umbrella windows are listed. All distance restraints were applied using the same force constant of 1000 kJ/mol. The distance at the minimum according to the PMF profile is supplied. Depending on the interface different simulation

times are required for equilibration as shown in Figure S22. The simulation time used for generating the PMFs are therefore listed for the different interfaces.

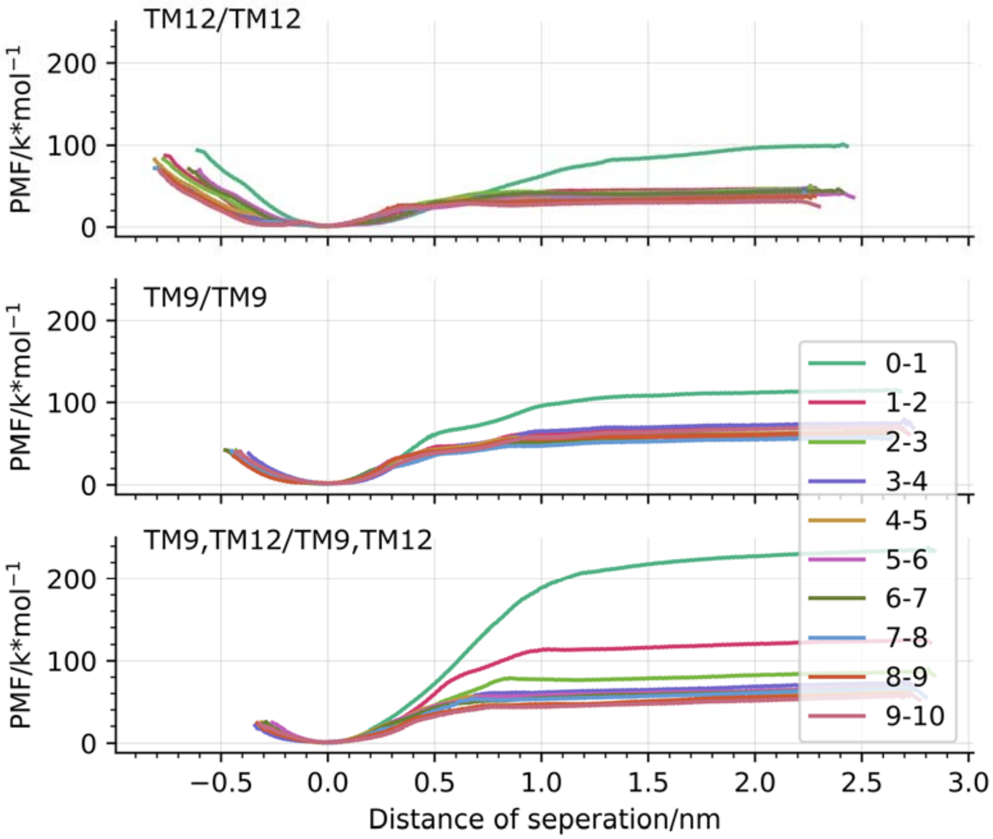

Figure S23: Time-evolution in μs time intervals of the different PMF profiles for the three hDAT-palm interfaces studied.

Interface	TM12/TM12	TM9/TM9	TM9,TM12/TM9,TM12	TM9,TM12/TM9,TM12 (hSERT)
ϕ1 (°)	-42.40	2.06	-32.09	-32.09
ϕ3 (°)	-42.40	2.06	-32.09	-32.09
ϕ1, ϕ3 force (kJ/mol)	300.00	300.00	300.00	300.00
distance force (kJ/mol)	1000.00	1000.00	1000.00	1000.00
distance at minimum (Å)	5.70	6.20	5.50	5.60
Simulation used for PMF calculation (μs)	2-10	1-10	3-10	3-10
Umbrella distance (Å)	4.90	5.70	5.30	5.20
	5.00	5.80	5.40	5.30
	5.10	5.90	5.50	5.40
	5.20	6.00	5.60	5.50
	5.30	6.10	5.70	5.60
	5.40	6.20	5.80	5.70
	5.50	6.30	5.90	5.80
	5.60	6.40	6.00	5.90
	5.70	6.50	6.05	6.00
	5.80	6.60	6.10	6.10
	5.90	6.70	6.20	6.15
	6.00	6.80	6.30	6.22
	6.10	6.90	6.40	6.31
	6.20	7.00	6.50	6.40
	6.30	7.10	6.60	6.51
	6.40	7.20	6.70	6.60
	6.50	7.30	6.80	6.70
	6.60	7.40	6.90	6.80
	6.70	7.50	7.00	6.90
	6.80	7.60	7.10	7.00
	6.90	7.70	7.20	7.10
	7.00	7.80	7.30	7.20
	7.10	7.90	7.40	7.30
	7.20	8.00	7.50	7.40
	7.30	8.10	7.60	7.50
	7.40	8.20	7.70	7.60
	7.50	8.30	7.80	7.70
	7.60	8.40	7.90	7.80
	7.70	8.50	8.00	7.90
	7.80	8.60	8.10	8.00

Table S2: Overview of : settings applied in the US-REMD hDAT-palm and hSERT simulations. Listed are the ϕ1 and ϕ3 angle values and their restraint force constant for the different interfaces. In addition, the distance restraints applied at the different umbrella windows are listed. All distance restraints were applied using the same force constant of 1000 kJ/mol. The distance at the minimum of the PMF's is shown. The distance at the minimum according to the PMF

profile is supplied. Depending on the interface different simulation times are required for equilibration as shown in Figure S23. The simulation time used for generating the PMFs are therefore listed for the different interfaces.

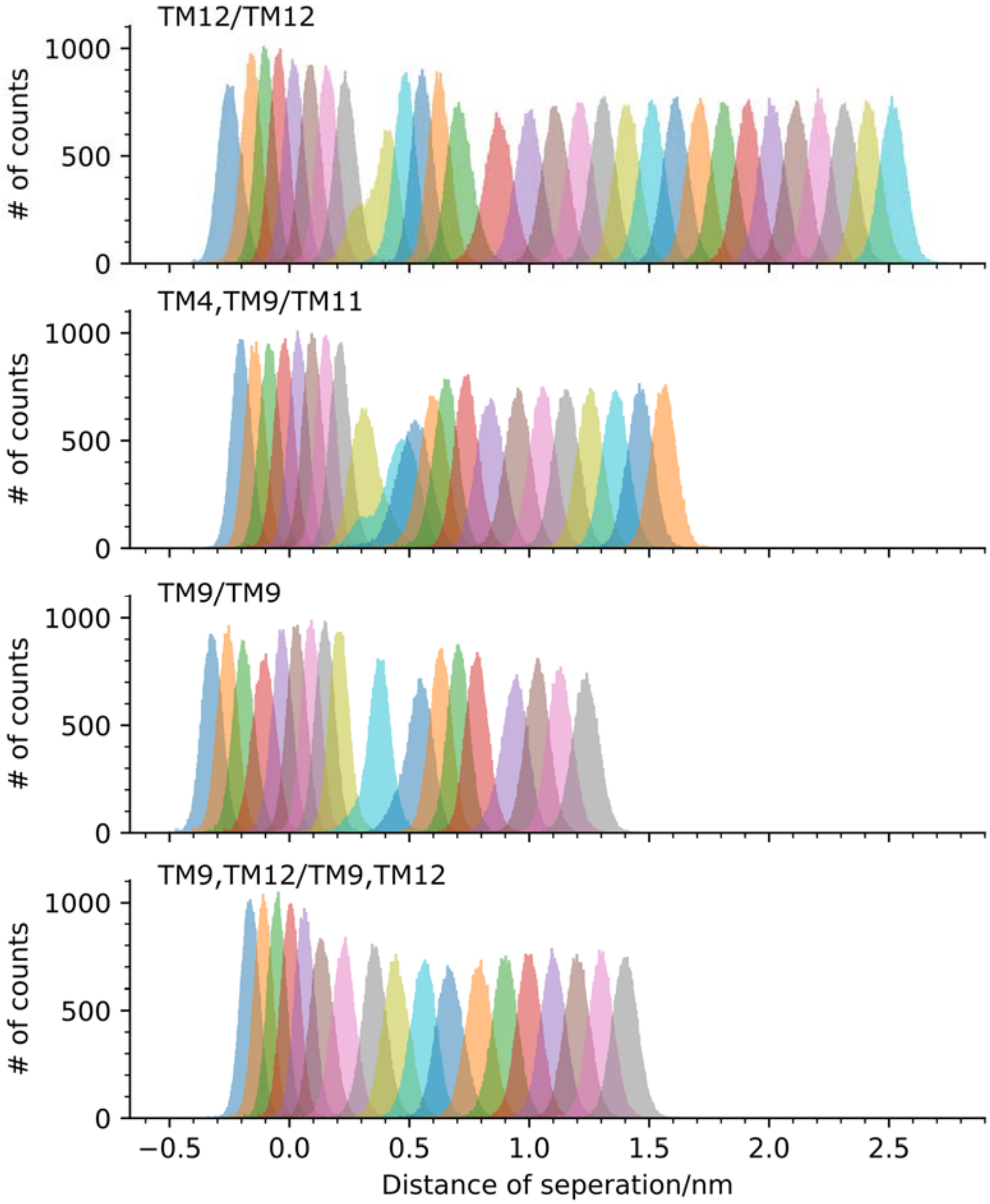

Figure S24: Histograms of the dimer separation distances in the different umbrella windows for the different hDAT interfaces.

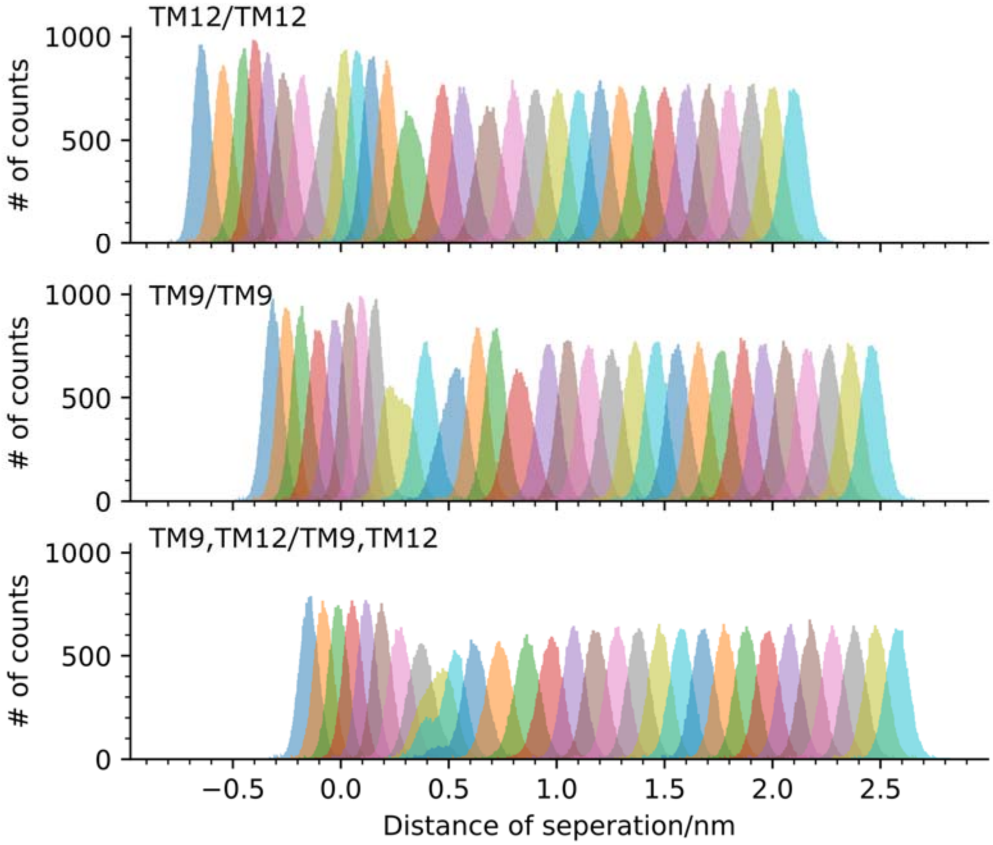

Figure S25: Histograms of the dimer separation distances in the different umbrella windows for the different hDAT-palm interfaces.

References

1 Zeppelin, T., Ladefoged, L. K., Sinning, S., Periole, X. & Schiøtt, B. A direct interaction of cholesterol with the dopamine transporter prevents its out-to-inward transition. *PLoS computational biology* **14**, e1005907 (2018).

2 Abraham, M. J. *et al.* GROMACS: High performance molecular simulations through multi-level parallelism from laptops to supercomputers. *SoftwareX* **1–2**, 19-25 (2015).

3 Berendsen, H. J. C., Van Der Spoel, D. & van Drunen, R. GROMACS: A message-passing parallel molecular dynamics implementation. *Comp. Phys. Commun.* **91**, 43-56 (1995).

4 Best, R. B. *et al.* Optimization of the Additive CHARMM All-Atom Protein Force Field Targeting Improved Sampling of the Backbone φ, ψ and Side-Chain χ1 and χ2 Dihedral Angles. *Journal of Chemical Theory and Computation* **8**, 3257-3273 (2012).

5 Foloppe, N. & MacKerell, A. D., Jr. All-atom empirical force field for nucleic acids: I. Parameter optimization based on small molecule and condensed phase macromolecular target data. *Journal of Computational Chemistry* **21**, 86-104 (2000).

6 Hess, B., Bekker, H., Berendsen, H. J. C. & Fraaije, J. G. E. M. LINCS: A Linear Constraint Solver for Molecular Simulations. *Journal of Computational Chemistry* **18**, 1463-1472 (1997).

7 Hess, B. P-LINCS: A Parallel Linear Constraint Solver for Molecular Simulation. *Journal of Chemical Theory and Computation* **4**, 116-122 (2008).

8 Miyamoto, S. & Kollman, P. A. SETTLE: an analytical version of the SHAKE and RATTLE algorithm for rigid water models. *Journal of computational chemistry* **13**, 952-962 (1992).

9 Lee, J. *et al.* CHARMM-GUI input generator for NAMD, GROMACS, AMBER, OpenMM, and CHARMM/OpenMM simulations using the CHARMM36 additive force field. *Journal of chemical theory and computation* **12**, 405-413 (2015).

10 Evans, D. J. & Holian, B. L. The nose–hoover thermostat. *The Journal of chemical physics* **83**, 4069-4074 (1985).

11 Parrinello, M. & Rahman, A. Polymorphic transitions in single crystals: A new molecular dynamics method. *Journal of Applied Physics* **52**, 7182-7190 (1981).

12 Berendsen, H. J. C., Postma, J. P., van Gunsteren, W. F., DiNola, A. & Haak, J. R. Molecular dynamics with coupling to an external bath. *J. Chem. Phys.* **81**, 3684-3690 (1984).

13 Steinbach, P. J. & Brooks, B. R. New Spherical-Cutoff Methods for Long-Range Forces in Macromolecular Simulation. *J. Comput. Chem.* **15**, 667-683 (1994).

14 Essmann, U. *et al.* A smooth particle mesh Ewald method. *The Journal of chemical physics* **103**, 8577-8593 (1995).

15 Beitz, E. TeXshade: shading and labeling of multiple sequence alignments using LaTeX2e. *Bioinformatics* **16**, 135-139 (2000).
